# Phase separation as a key mechanism in plant development, environmental adaptation, and abiotic stress response

**DOI:** 10.1016/j.jbc.2025.108548

**Published:** 2025-04-24

**Authors:** Karina C. Pougy, Bruna A. Brito, Giovanna S. Melo, Anderson S. Pinheiro

**Affiliations:** Department of Biochemistry, Institute of Chemistry, Federal University of Rio de Janeiro, Rio de Janeiro, RJ, Brazil

**Keywords:** condensate, growth, phase, plant, separation, stress

## Abstract

In memoriam of Professor Anderson de Sá Pinheiro, principal investigator at the Laboratory of Molecular Biology (LabMol) at the Institute of Chemistry, Federal University of Rio de Janeiro (UFRJ). Prof. Pinheiro passed away prematurely at the age of 44, on March 1, 2025. Prof. Pinheiro was a distinguished figure in the fields of biochemistry and structural biology in Brazil. He earned his bachelor's degree in Pharmacy in 2000, his master's degree in 2003, and his Ph.D. in 2007, all in Biological Chemistry at UFRJ. He continued his academic journey with postdoctoral research at Brown University in the United States (2007–2011). Upon returning to Brazil, he became an Associate Professor in the Department of Biochemistry at UFRJ (2011–2025). He led the Laboratory of Molecular Biochemistry (LaBMol), focusing on the study of RNA-binding proteins related to cancer and neurodegenerative disorders, as well as plant responses to abiotic stress. His research followed two main fronts—analyzing protein structures and dynamics using solution NMR spectroscopy and investigating the relationship between structural features and liquid–liquid phase separation, along with its role in protein function. Beyond research, Prof. Anderson was deeply committed to education, mentoring numerous students and contributing to various academic committees. During his brief but impactful career, he made significant contributions to the structural biology community, serving as President of the Brazilian Association of Nuclear Magnetic Resonance Users (AUREMN) and as Scientific Director of the Brazilian Biophysical Society (SBBf). This review marks Professor Pinheiro's 50th published article. His untimely passing is a profound loss to the scientific community, but his legacy endures through his scientific contributions and the many lives he has touched.

**Figure:**
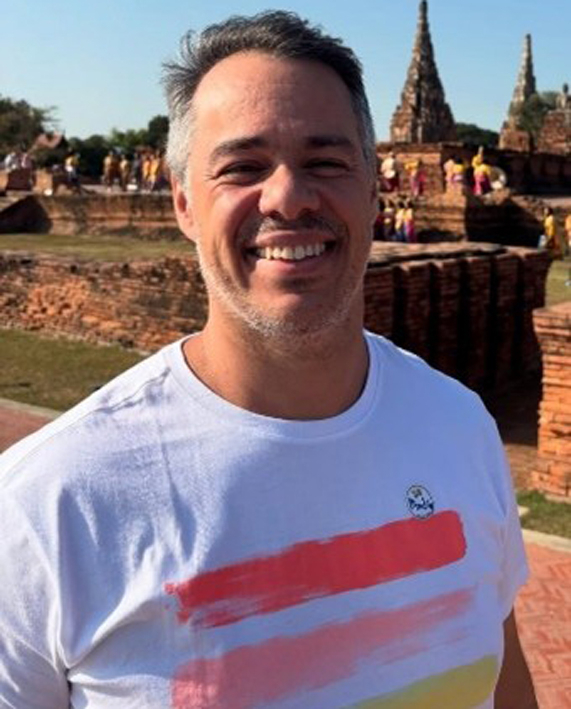
This tribute to the late Prof. Anderson Pinheiro was previously allowed by JBC with the agreement of the associate editor Dr. Karin Musier-Forsyth.

This tribute to the late Prof. Anderson Pinheiro was previously allowed by JBC with the agreement of the associate editor Dr. Karin Musier-Forsyth.

Liquid–liquid phase separation is a fundamental biophysical process in which biopolymers, such as proteins, nucleic acids, and their complexes, spontaneously demix into distinct coexisting phases. This phenomenon drives the formation of membraneless organelles—cellular subcompartments without a lipid bilayer that perform specialized functions. In plants, phase-separated biomolecular condensates play pivotal roles in regulating gene expression, from genome organization to transcriptional and post-transcriptional processes. In addition, phase separation governs plant-specific traits, such as flowering and photosynthesis. As sessile organisms, plants have evolved to leverage phase separation for rapid sensing and response to environmental fluctuations and stress conditions. Recent studies highlight the critical role of phase separation in plant adaptation, particularly in response to abiotic stress. This review compiles the latest research on biomolecular condensates in plant biology, providing examples of their diverse functions in development, environmental adaptation, and stress responses. We propose that phase separation represents a conserved and dynamic mechanism enabling plants to adapt efficiently to ever-changing environmental conditions. Deciphering the molecular mechanisms underlying phase separation in plant stress responses opens new avenues for biotechnological strategies aimed at engineering stress-resistant crops. These advancements have significant implications for agriculture, particularly in addressing crop productivity in the face of climate change.

## General principles of phase separation

Eukaryotic cells are highly organized systems that compartmentalize biochemical reactions and molecular processes in distinct organelles. While many of these compartments are membrane-bound, cells also harbor membraneless organelles (MLOs), which are dynamic, functional compartments formed without a lipid bilayer (1, 2, 3, 4). MLOs are found in both the cytoplasm and the nucleus and, in some cases, even inside specific membrane-bound organelles (1, 2, 3, 4). Examples include stress granules (SGs) (5), processing bodies (PBs) (6), and Balbiani bodies (7) in the cytoplasm, as well as the nucleolus (8), Cajal bodies (9), and paraspeckles (10) in the nucleus. As research has advanced, the list of MLOs continues to expand, underscoring their importance across diverse cellular contexts.

MLOs are typically formed through liquid-liquid phase separation (LLPS), a biophysical process in which a supersaturated solution of biomolecules—proteins, nucleic acids, or their complexes—spontaneously demixes into two distinct coexisting phases: a condensed phase enriched in biomolecules and a dilute phase that is biomolecule-poor (1, 2, 3, 4). This process often results in biomolecular condensates that retain liquid-like properties, such as a spherical morphology, fusion, fission, and rapid molecular exchange with the surrounding environment (1, 2, 3, 4, 11). Evidence of their fluidity is frequently observed through techniques such as fluorescence recovery after photobleaching, where dynamic molecular exchange within the condensate or with the dilute phase is demonstrated by rapid fluorescence recovery (1, 2, 3, 4, 11, 12).

A key driver of LLPS is multivalency, the capacity of biomolecules to establish dense networks of intramolecular and intermolecular interactions (1, 2, 3, 4, 13). Proteins that undergo phase separation often possess multiple interaction motifs, enabling a high degree of intermolecular connectivity. Two main classes of phase-separating proteins exist: multidomain proteins, in which folded domains are linked by flexible regions, and intrinsically disordered proteins (IDPs), which either lack a defined structure throughout their entire sequence or contain intrinsically disordered regions (IDRs) (1, 2, 3, 4, 13). In the first case, interactions occur either between the folded domains or with the flexible linkers. In the second case, interactions involve short linear motifs in disordered regions, which often manifest as degenerate sequences or clusters of alternating charges, collectively known as low-complexity domains (LCDs) (1, 4, 14). IDPs are particularly prone to LLPS because their sequence bias toward certain amino acids promotes key molecular interactions. Their phase behavior is driven by a combination of electrostatic interactions, π–π stacking between aromatic residues, cation–π interactions between positively charged and aromatic residues, hydrophobic interactions, and hydrogen bonding (4, 14). Prominent short linear motifs in IDPs include RGG motifs, which are rich in arginine and glycine (15), and prion-like domains (PrLDs), which are enriched in noncharged polar residues such as glutamine, asparagine, proline, serine, and glycine (16). These domains may also contain sparsely distributed aromatic amino acids, such as tyrosine and phenylalanine (16). Such motifs are often found in RNA-binding proteins (RBPs), further increasing their propensity for phase separation (4, 14, 15, 16, 17).

The sticker-and-spacer model offers a framework for understanding how amino acid composition influences phase separation. Stickers are residues that mediate adhesive intermolecular interactions, whereas spacers separate stickers and modulate condensate material properties (18, 19, 20). For example, arginine and tyrosine are strong stickers essential for determining the saturation concentration for phase separation. In contrast, glycine, serine, glutamine, asparagine, and proline serve as spacers, with their flexibility influencing whether the condensate adopts a more fluid or solid-like state (18, 19, 20). Over time, biomolecular condensates may transition from liquid to gel-like or solid phases in a process known as aging, which is implicated in diseases associated with protein aggregation (17, 21, 22). RNA also plays a pivotal role in LLPS, acting as a multivalent and flexible polymer that can drive phase separation (23) or modulate protein condensation (24). RNA molecules can either increase or suppress phase separation by forming extensive interaction networks with RBPs, further diversifying the functional and structural properties of condensates (24).

Biomolecular condensates perform a wide array of functional roles (12, 25, 26). Within the condensed phase, high local concentrations of proteins, nucleic acids, and small molecules increase reaction rates, facilitating the formation of signaling complexes (27), nucleation-dependent polymerization (27, 28), and enzymatic catalysis (29, 30). Condensates accelerate biochemical reactions by co-concentrating enzymes and substrates while also modulating the conformational dynamics of molecules (30). Conversely, they can inhibit reactions by sequestering (27, 31) or excluding (27) specific molecules from their sites of action. In addition, condensates provide a controlled environment for protein folding (32) and RNA stabilization (33), preventing aggregation. LLPS organizes the cellular architecture by forming distinct compartments that spatially segregate biochemical processes. This is exemplified by the nucleolus, where phase separation mediates ribosome biogenesis through the coexistence of multiple dense phases (8). Furthermore, condensates play a role in synaptic organization (34), autophagosome formation (35), molecular filtration at nuclear pores (36), and genome organization (37, 38). Emerging evidence also suggests that condensates can exert mechanical forces, remodeling cellular structures, including membranes (39). At the cellular level, condensates define specialized compartments that enable the precise localization of biomolecules. They also function as concentration buffers, maintaining a constant protein concentration in the dilute phase by increasing their size and number in response to elevated total protein levels (12, 26). Given their sensitivity to environmental conditions such as pH, ionic strength, and temperature, condensates provide a rapid, adaptive, and reversible mechanism for sensing and responding to stress (22, 40, 41, 42). Collectively, these findings highlight the functional versatility of biomolecular condensates in coordinating cellular homeostasis, signal transduction, and stress adaptation. The formation, biophysical properties, and functional roles of biomolecular condensates have been extensively investigated across diverse biological systems. While this review provides a concise summary of these fundamental concepts, a detailed discussion is beyond its scope. For a more in-depth examination of these topics, readers are referred to competent reviews on condensate formation mechanisms (1, 2, 3, 4, 12, 14, 22), the biophysical principles underlying phase separation (43, 44, 45, 46), and the functional implications of biomolecular condensates (12, 25, 26, 30, 42, 47, 48, 49).

In plants, LLPS is emerging as a fundamental mechanism for integrating environmental signals into physiological responses, crucial for growth, development, and stress adaptation. Plants, as sessile organisms, face constant exposure to environmental stressors such as extreme temperatures, drought, salinity, and nutrient scarcity—challenges exacerbated by climate change (50). Given their significance in global food security and biomass production, equipping plants with the ability to withstand abiotic stresses is essential. LLPS provides a versatile strategy for rapid, dynamic responses to environmental changes, offering new biotechnological avenues to enhance crop resilience and yield.

**Figure 1:**
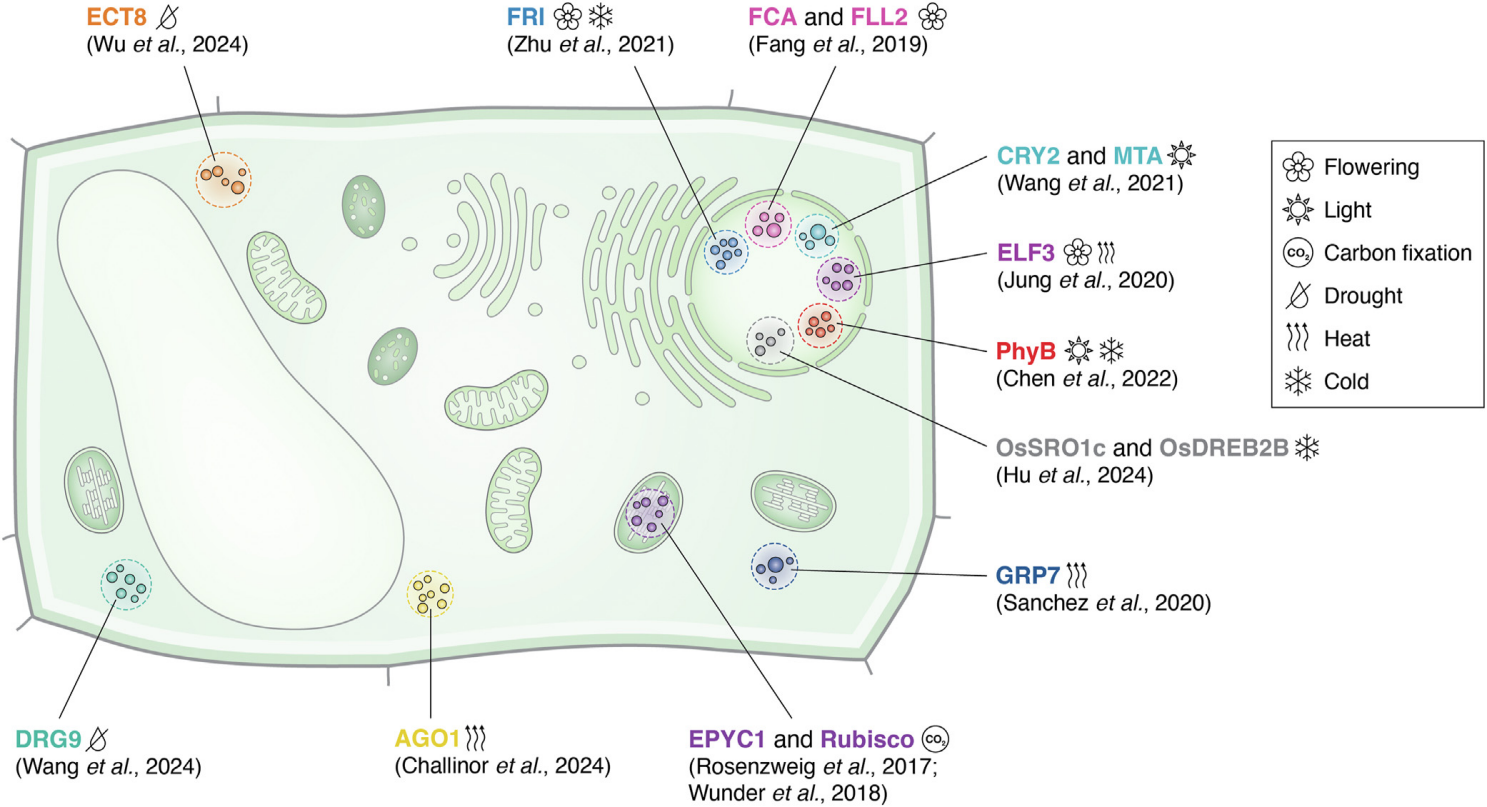
**Representative biomolecular condensates in plants.** Schematic representation of a plant cell highlighting major organelles alongside examples of biomolecular condensates. Cytoplasmic and nuclear condensates are depicted as clusters of color-coded circles, each labeled with the name of the protein undergoing phase separation, an icon representing the associated biological process or external stimulus, as indicated in the figure legend, and the relevant citation. *Flowering*: FCA (*pink*) co-condenses with FLL2 (*pink*) to regulate flowering (flower icon) *via* the autonomous pathway. In response to cold (snowflake icon), FRIGIDA (FRI) (*light blue*) undergoes phase separation to regulate flowering through the vernalization pathway. *Carbon fixation*: EPYC1 (*dark purple*) co-condenses with RuBisCO (*dark purple*) to form the pyrenoid within chloroplasts, increasing carbon fixation (CO_2_ icon). *Light signaling*: In response to *blue* light (sun icon), CRY2 (*light cyan*) co-condenses with MTA (*light cyan*) to promote m^6^A RNA methylation. In response to *red* light (sun icon) or low temperatures (snowflake icon), PhyB forms nuclear photobodies through LLPS, facilitating photomorphogenesis. *Heat stress:* In response to heat (curved arrows icon), ELF3 undergoes nuclear phase separation to regulate flowering (flower icon) and hypocotyl elongation. In addition, the RNA-binding proteins GRP7 (*dark blue*) and AGO1 (*yellow*) undergo cytoplasmic phase separation under heat stress, facilitating heat stress adaptation. *Cold stress*: In response to cold (snowflake icon), OsSRO1c (*light gray*) co-condenses with the transcription factor OsDREB2B (*dark gray*) in the nucleus, regulating the cold stress response in rice. *Drought stress*: Under drought conditions (crossed drop icon), DRG9 (*dark cyan*) and the m^6^A reader ECT8 (*orange*) undergo cytoplasmic phase separation to enhance the drought stress response.

This review explores recent advances in understanding biomolecular condensation in plants, focusing on its diverse roles in development, environmental adaptation, and stress responses. We begin by examining the role of phase separation in the regulation of gene expression, including chromatin condensation, m^6^A RNA methylation, and the processing of miRNAs and siRNAs. Next, we explore how phase separation contributes to key developmental processes, such as flowering time regulation, circadian rhythm, and photosynthesis. Finally, we address the critical role of phase separation in plant responses to various abiotic stresses, including heat, cold, drought, salinity, and nutrient deficiency. Figure 1 illustrates representative examples of biomolecular condensates in plants, as discussed in this review. In addition, Table 1 summarizes all the phase-separating proteins covered in this review, along with the biomolecular assemblies they form, their biochemical mechanisms, and functional outcomes.

**Table 1:** Summary of biomolecular condensates in plants discussed in this review, along with their respective biochemical mechanisms and physiological outcomes

Condensate	Assembly Protein(s)	Biochemical mechanism	Physiological function	References
Heterochromatin foci	ADCP1, HMGA	ADCP1 binds H3K9me2-marked chromatin and undergoes phase separation, facilitated by HMGA co-condensation	Heterochromatin formation and TE silencing	(55, 56)
MtSUVR2 condensate	MtSUVR2	MtSUVR2 condensates facilitate H3K9me2 deposition and recruit MtRAD51 recombinase	Heterochromatin formation and DNA damage repair in euchromatic regions	(57)
Heterochromatin foci	Histone H1	H1 phase separates through its C-terminal IDR, promoting nucleosome condensation	Heterochromatin formation	(58)
Chromatin condensate	Histone H2B.8	H2B.8 aggregates transcriptionally inactive AT-rich chromatin into phase-separated condensates, promoting nuclear compaction without suppressing transcription	Sperm cell chromatin condensation	(59)
CPSF30-L condensate	CPSF30-L	CPSF30-L phase separates upon binding m^6^A-modified mRNAs, promoting their 3′-proximal polyadenylation	Regulation of flowering time and ABA signaling	(66)
SiYTH2 condensate	SiYTH2	SiYTH2 phase separates upon binding m^6^A-modified mRNAs, inhibiting their translation	Regulation of volatile compound biosynthesis	(67)
P-body/Stress granule	ECT1	ECT1 undergoes phase separation into SGs and PBs, recruiting m^6^A-modified mRNAs and promoting their decay	Regulation of salicylic acid–dependent immune response	(68)
Stress granule	EHD6, YTH07	EHD6 and YTH07 phase separate into SGs, sequestering m^6^A-modified *OsCOL4* mRNA and inhibiting its translation	Flowering time control	(69)
Dicing body (D-body)	SE	SE phase separates, recruiting DCL1 and HYL1 for pri-miRNA processing	miRNA biogenesis	(74)
SE-MTB condensate	SE	SE condensates stabilize MTB and FIP37, enhancing m^6^A deposition on pri-miRNAs	Cotranscriptional pri-miRNA processing	(78, 79)
siRNA body	SGS3	SGS3 phase separates and recruits siRNA processing factors, including RDR6, DCL2, DCL4, AGO1, and AGO7	Epigenetically activated siRNA (easiRNA) biogenesis	(81, 82)
FCA condensate	FCA, FLL2	FCA/FLL2 condensates recruit polyadenylation factors, enhancing *COOLAIR* 3′-end processing and inhibiting *FLC* transcription	Flowering time control *via* the autonomous pathway	(84)
FRI condensate	FRI	FRI phase separates upon cold exposure, sequestering transcriptional activators and repressing *FLC* transcription	Flowering time control *via* the vernalization pathway	(85)
EMB1579 condensate	EMB1579	EMB1579 condensates recruit MSI4-DDB1-CUL4 and CLF-PRC2 to regulate H3K27me3 levels, repressing *FLC*	Flowering time regulation	(86)
HRLP condensate	HRLP	HRLP forms nuclear condensates that regulate *FLC* splicing and R-loop formation, repressing *FLC*	Flowering time regulation	(87)
DCP5-SSF condensate	DCP5, SSF	DCP5 and SSF condensates bind *FLC* chromatin, decreasing RNA polymerase II enrichment and repressing *FLC*	Flowering time regulation	(88)
CO condensate	CO	Upon extended daylight exposure, CO co-condenses with NF-YB2 and NF-YC9, enhancing *FT* transcription	Flowering time control *via* the photoperiod pathway	(89)
TMF condensate	TMF	Elevated H_2_O_2_ levels oxidize TMF cysteines, enhancing its phase separation and repressing *AN*	Flowering time regulation	(110)
TFAM condensate	TFAMs	TFAMs assemble into heterotypic condensates that modulate *AN* expression	Flowering time regulation	(111)
Pyrenoid	EPYC1, RuBisCO	EPYC1 and RuBisCO condensates concentrate CO_2_, saturating RuBisCO	CO_2_ fixation	(114, 116)
Photobody	CRY2	Blue light induces CRY2 phase separation, recruiting m^6^A writers, MTA, MTB, and FIP37, enhancing m^6^A methylation and stabilizing circadian oscillator mRNAs	Circadian rhythm regulation	(63)
Photobody	CRY2, FIO1, SPA1	Blue light triggers CRY2-FIO1-SPA1 phase separation, promoting m^6^A methylation and translation of mRNAs encoding chlorophyll homeostasis regulators	Photosynthesis regulation	(64)
Photobody	CRY1, CRY2	Blue light triggers CRY1/2 condensation, accumulating MAC3A/3B, which bind chromatin and counteract HY5, promoting hypocotyl elongation	Skotomorphogenesis	(125)
Photobody	PhyB	Red light induces PhyB condensation, recruiting PIF transcription factors and enabling switch-control of PhyB signaling	Photomorphogenesis	(129, 130)
Photobody	PhyB	Under low temperatures, PhyB condenses in the nucleus, sequestering transcriptional machinery and regulating hypocotyl elongation	Thermomorphogenesis	(129)
Photobody	PhyB	PhyB undergoes phase separation in response to red light, stabilizing PIF5 and preventing its degradation	Photomorphogenesis	(131)
Photobody	PhyB	PhyB undergoes phase separation in response to red light, sequestering PIF7 and repressing the expression of PIF7-target genes	Photomorphogenesis	(132)
Photobody	PhyA, TZP	In response to far-red light, PhyA and TZP condense and recruit PPKs, which phosphorylate PhyA and TZP, resulting in PhyA degradation	Photomorphogenesis	(134)
ELF3 condensate	ELF3	At elevated temperatures, ELF3 undergoes condensation and sequesters the evening complex, preventing its interaction with chromatin	Thermomorphogenesis	(142)
Stress granule	ALBA4, ALBA5, ALBA6	Under heat stress, ALBA proteins undergo phase separation into SGs, sequestering HSF mRNAs to prevent their degradation	Heat stress response	(148)
Stress granule	RBGD2, RBGD4	Under heat stress, RBGD2 and RBGD4 condense into SGs, sequestering mRNAs that encode HSFs and oxidative stress regulators	Heat stress response	(149)
Stress granule	GRP7	Under heat stress, GRP7 condenses and sequesters mRNAs, translation initiation factors, and cold shock proteins into SGs, inhibiting the translation of heat-responsive mRNAs	Heat stress response	(150)
Stress granule	AGO1	Under heat stress, AGO1 condenses and accumulates in SGs, along with siRNA body components and mRNA decay proteins	Heat stress response	(151)
CP29A condensate	CP29A	At low temperatures, CP29A forms condensates inside chloroplasts, regulating chloroplast RNA splicing and translation	Cold adaptation	(156)
OsRO1c condensate	OsRO1c	Under cold stress, OsSRO1c undergoes condensation and recruits OsDREB2B, enhancing its transcriptional activity	Cold stress response	(157)
Stress granule	DRG9	Under drought stress, DRG9 undergoes phase separation into SGs, stabilizing *OsNCED4* mRNA and promoting ABA biosynthesis	Drought stress response	(160)
SiYTH1 condensate	SiYTH1	Under drought stress, SiYTH1 undergoes phase separation, stabilizing specific m6A-methylated transcripts and enhancing ABA signaling	Drought stress response	(161)
Stress granule	ECT8	Under drought stress, ECT8 undergoes phase separation into SGs, sequestering m^6^A-modified mRNAs, including the ABA receptor PYL7, and regulating ABA signaling	Drought stress response	(162)
SEU condensate	SEU	Under osmotic stress, SEU forms nuclear condensates that promote the expression of stress-responsive genes	Osmotic stress response	(163)
DCP5-enriched osmotic stress granule (DOSG)	DCP5	Under osmotic stress, DCP5 undergoes phase separation into DOSGs, sequestering specific mRNAs and reprogramming the transcriptome toward stress-responsive mRNAs	Osmotic stress response	(164)
FLO1 condensate	FLOE1	FLOE1 undergoes phase separation in response to hydration, promoting germination	Germination	(169)
STM condensate	STM	Under salt stress, STM undergoes condensation, promoting its interaction with MED8, which enhances its transcriptional activity and stimulates shoot branching	Salt stress response	
RALF-pectin condensates	RALF, pectin	Under salt and heat stress, RALF interacts with pectin and undergoes extracellular condensation, recruiting the FER–LGG1 complex, which amplifies receptor endocytosis and activates signaling pathways	Salt and heat stress response	(172)
MED19a condensate	MED19a	Under nitrogen deficiency, MED19a undergoes phase separation, recruiting ORE1 to induce senescence-related genes	Nitrogen deficiency response	(177)

## Phase separation in gene expression regulation

### Chromatin status

Eukaryotic genomes are organized into complex three-dimensional structures that enable spatial compartmentalization of genomic activities in the nucleus (51). Chromatin, which is composed of genomic DNA bound to histones and nonhistone proteins, is broadly categorized into euchromatin and heterochromatin, with the latter further divided into constitutive and facultative subtypes. These chromatin states differ markedly in their structural organization, epigenetic signatures, and functional roles (52, 53). Euchromatin is characterized by a loosely packed, open conformation enriched in transcriptionally active histone marks, such as trimethylation of histone H3 at lysine 4 (H3K4me3) and acetylation of histone 3 at lysine 27 (H3K27ac) (52, 53). Conversely, constitutive heterochromatin is tightly compacted and transcriptionally repressive and is primarily localized to centromeric and telomeric regions. It is enriched with repetitive DNA and transposable elements (TEs) and is maintained by distinct epigenetic signatures, including H3K9me2/3 and extensive DNA methylation (52, 53). Facultative heterochromatin, on the other hand, is more dynamic, transitioning between repressive and active states. It is defined by the presence of H3K27me3, a repressive histone mark deposited by Polycomb group complexes, and it plays a role in regulating tissue-specific and developmental gene expression (52, 53).

Constitutive heterochromatin is integral to plant biology, safeguarding genome stability, repressing TEs, and ensuring proper nuclear organization (53). Formation of the heterochromatin is driven by multiple mechanisms, including the dimethylation of histone H3 at lysine 9 (H3K9me2), which is catalyzed by members of the SU(VAR)3-9 homolog family of methyltransferases (54). H3K9me2 serves as a key marker of silenced chromatin, recruiting chromatin reader proteins to facilitate compaction (53). Cytosine methylation in DNA across CG, CHG, and CHH contexts (where C represents cytosine, G represents guanine, and H denotes any nucleotide except guanine) further reinforces silencing, with the RNA-directed DNA methylation (RdDM) pathway playing a significant role (53). Small RNAs and long noncoding RNAs guide chromatin-modifying enzymes to specific loci, whereas ATP-dependent chromatin remodelers increase nucleosome stability in heterochromatic regions (53). Recent evidence highlights LLPS as a fundamental mechanism in heterochromatin assembly and maintenance. Multiple heterochromatin components, including methylation mark readers, writers, histones, and their variants, form phase-separated condensates, contributing to chromatin compaction (55, 56, 57, 58, 59).

Weak, multivalent interactions between DNA, histones, and phase-separating proteins drive the formation of liquid-like condensates, contributing to chromatin compartmentalization (49). This process operates through two primary mechanisms. First, chromatin-associated proteins or complexes engage in multivalent interactions with DNA or nucleosomal arrays, with chromatin actively participating in condensate formation. These interactions are further modulated by DNA sequence features and epigenetic modifications (49). In the second mechanism, chromatin-associated proteins containing IDRs undergo phase separation through weak self-associating interactions, with chromatin primarily serving as a binding scaffold (49). These mechanisms are not mutually exclusive and can function cooperatively, enhancing phase separation and chromatin compaction.

**Figure 2:**
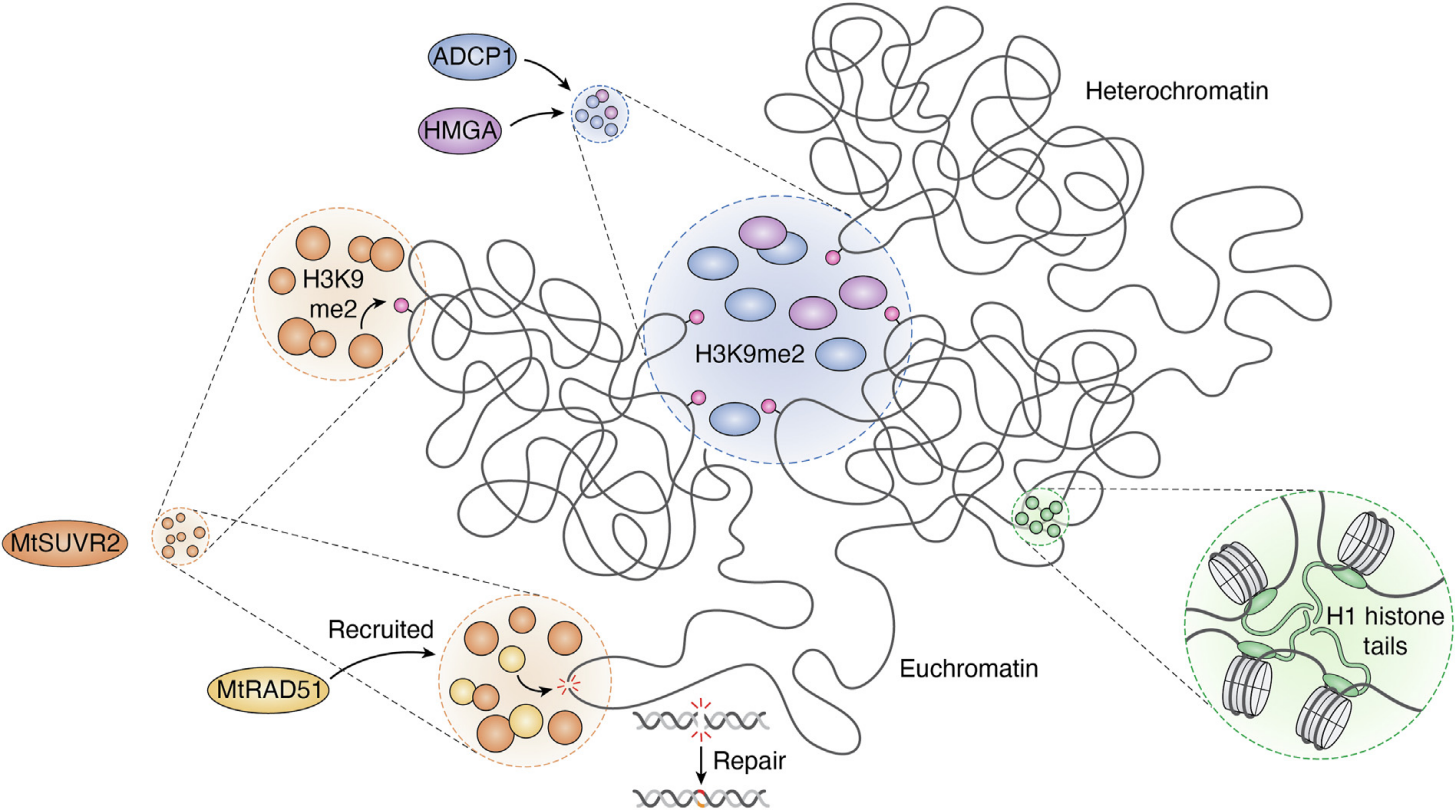
**Phase separation as a mechanism for chromatin organization.** The Agenet domain-containing protein, ADCP1 (*blue*), specifically recognizes the silenced chromatin mark H3K9me2 (*pink sphere*), undergoing phase separation. In addition, the high mobility group protein, HMGA (*purple*), co-condenses with ADCP1, promoting the formation of heterochromatin foci. The methyltransferase MtSUVR2 (*dark orange*) undergoes phase separation to catalyze H3K9me2 (*pink sphere*) deposition. Furthermore, MtSUVR2 condensates recruit the recombinase MtRAD51 (*light orange*), facilitating DNA double-strand break repair. Finally, histone H1 (*green*) undergoes phase separation through its C-terminal intrinsically disordered tail, contributing to chromatin compaction.

In plants, the Agenet domain-containing protein ADCP1 specifically recognizes H3K9me2 (60). Loss-of-function mutations in *adcp1* result in significant chromatin decondensation, reduced H3K9me2 levels, decreased CHG/CHH DNA methylation, and elevated TE expression (55, 60). ADCP1 forms liquid-like condensates in the presence of H3K9me2-marked nucleosome arrays, suggesting that phase separation underlies its role in genome organization (Fig. 2) (55). The activity of ADCP1 is critically dependent on its interaction with the high mobility group protein HMGA, which enhances the phase separation capacity of ADCP1. Through co-condensation, ADCP1 and HMGA facilitate heterochromatin aggregation by binding to H3K9me2-marked chromatin, repressing TE activity (Fig. 2) (56).

In addition to histone-methyl readers, writers also contribute to LLPS-driven chromatin regulation. In *Medicago trunculata*, the histone methyltransferase MtSUVR2 catalyzes H3K9me2 deposition and undergoes phase separation *via* its IDR (57). MtSUVR2 condensates recruit the recombinase MtRAD51, directing it to DNA damage sites within euchromatin (57). In response to double-strand breaks, MtSUVR2 mediates H3K9me2 methylation, promoting heterochromatin formation and enhancing genomic resistance to DNA damage (57). Concurrently, MtSUVR2 drives the phase separation of MtRAD51, supporting efficient double-strand break repair in euchromatic regions (Fig. 2) (57).

The linker histone H1 is another key player in heterochromatin condensation, promoting interactions within and between pericentromeric regions (58). H1 compacts chromatin into highly condensed foci through a phase separation mechanism that relies on its C-terminal IDR (C-IDR) (Fig. 2). Remarkably, a minimal construct comprising the C-IDR and a short segment of the globular domain is sufficient to aggregate chromatin and partially rescue the phenotypic defects observed in *h1* mutants (58). The C-IDR is also essential for H1 localization in heterochromatin and its key functions, including nucleosome spacing and DNA methylation (58). These findings highlight the importance of C-IDR–driven phase separation in H1-mediated heterochromatin foci formation and genome organization.

In flowering plant sperm cells, euchromatin undergoes significant compaction while remaining transcriptionally active (59). Unlike animals and nonseed plants, which utilize protamines to condense sperm chromatin and silence transcription (61), flowering plants rely on the histone variant H2B.8. This variant contains a long, disordered N-terminal tail that drives euchromatin compaction *via* LLPS (59). H2B.8 preferentially associates with AT-rich euchromatic TEs lacking H3K9me2 while preserving transcriptional activity (59). Interestingly, H2B.8-mediated condensation of euchromatic TEs contributes to heterochromatin dispersion in pericentromeric regions (59). This mechanism balances chromatin compaction with transcriptional activity, allowing the expression of genes essential for successful fertilization.

### m^6^A RNA methylation

N^6^-methyladenosine (m^6^A) is the most prevalent mRNA modification in eukaryotes. The levels of m^6^A methylation are dynamically regulated by the interplay of m^6^A writers (methyltransferases), erasers (demethylases), and readers (RNA-binding proteins) (62). In plants, the m^6^A writer complex comprises several components, including MTA, MTB, FIP37, virilizer, Hakai, Hakai-interacting zinc finger protein 2 (H1Z2), FIONA1, and METTL5 (62). The erasers consist of proteins from the ALKB family of dioxygenases, such as ALKBH9B, ALKBH9C, and ALKBH10B (62). The readers encompass proteins such as evolutionarily conserved C-terminal region proteins (ECT1 to ECT11) and cleavage and polyadenylation specific factor 30 (CPSF30). m^6^A readers typically share a conserved domain architecture characterized by a C-terminal YT521-B homology (YTH) domain, which contains an aromatic cage essential for m^6^A binding and an N-terminal PrLD-containing IDR (62). In plants, m^6^A modification plays pivotal roles in diverse developmental processes, including embryogenesis, organogenesis, and flowering, as well as in growth, environmental adaptation, and stress responses (62).

Increasing evidence suggests that m^6^A writers, erasers, and readers undergo LLPS to form dynamic, liquid-like granules with specialized functions (63, 64, 65, 66, 67, 68, 69, 70, 71). For instance, m^6^A writers, such as MTA, MTB, FIP37, and FIO1, co-condense with photoactivated photoreceptors (63, 64), as discussed later in this review. Similarly, the m^6^A eraser ALKBH9B localizes to phase-separated cytoplasmic granules, such as PBs and siRNA bodies (65). Notably, m^6^A readers often undergo LLPS, driven by interactions between their YTH domains and m^6^A-modified RNA and further facilitated by their N-terminal IDRs (66, 67, 68, 69, 70, 71). CPSF30-L, the longer isoform of the key polyadenylation factor CPSF30, illustrates this phenomenon. CPSF30-L binds m^6^A-modified mRNAs, inducing phase separation and the formation of liquid-like nuclear condensates (66). CPSF30-L preferentially recognizes far-upstream elements in the 3′-UTRs of target mRNAs, such as *SOC1*, *RPN10*, and *FYVE1* (66). This recognition promotes proximal polyadenylation, which enhances mRNA stability, thereby regulating key physiological processes, including flowering time and abscisic acid (ABA) signaling (66). LLPS-driven condensates may selectively enrich m^6^A-modified transcripts, facilitating their recognition by CPSF30-L and promoting the preferential selection of proximal polyadenylation sites. This spatial compartmentalization likely optimizes the efficiency and specificity of alternative polyadenylation.

In addition to alternative 3′-end processing, m^6^A-mRNA–induced condensates formed by m^6^A readers are crucial for translational regulation. The m^6^A reader SiYTH2 undergoes phase separation upon binding to m^6^A-modified mRNAs, such as *SiHPL* and *SiCCD1B*, inhibiting their translation and reducing the synthesis of volatile aromatic compounds in tomatoes (67). Similarly, the m^6^A reader ECT1 modulates the salicylic acid–dependent plant response to bacterial infection through phase separation. ECT1 forms cytoplasmic condensates that recruit m^6^A-modified mRNAs, promoting their decay and attenuating the salicylic acid–mediated plant stress response (68). In rice, the RNA-binding protein EARLY HEADING DATE 6 (EHD6) interacts with the m^6^A reader YTH07, enhancing its binding affinity for m^6^A-modified mRNAs (69). This interaction promotes the recruitment of YTH07 to cytoplasmic liquid-like granules that colocalize with SGs (69). The EHD6-YTH07 condensates sequester the m^6^A-modified *OsCOL4* transcript, suppressing its translation and reducing OsCOL4 protein levels, ultimately accelerating flowering (69). These findings support a model in which polymethylated RNA acts as a molecular scaffold, enabling multivalent interactions with m^6^A readers through their YTH domains. These interactions promote the assembly of the N-terminal PrLDs in m^6^A readers, driving LLPS (70, 71). The resulting m^6^A–mRNA–reader complexes partition into phase-separated subcellular compartments, such as SGs, regulating translational efficiency (70, 71).

### miRNA processing

**Figure 3:**
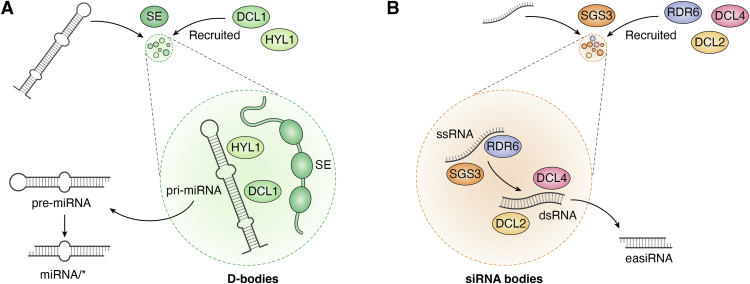
**Phase separation and RNA processing.***A*, miRNA maturation in phase-separated Dicing bodies (D-bodies). The disordered zinc finger protein SERRATE (SE) (*dark green*) forms condensates that recruit DICER-LIKE 1 (DCL1) (*medium green*), (HYPONASTIC LEAVES 1) HYL1 (*light green*), and pri-miRNAs. HYL1 and DCL1 collaborate to process pri-miRNA into pre-miRNA, which is subsequently converted into the mature miRNA/∗ duplex. *B*, siRNA biogenesis in phase-separated siRNA bodies. SUPPRESSOR OF GENE SILENCING 3 (SGS3) (*dark orange*) forms condensates that recruit various components, including RNA-DEPENDENT RNA POLYMERASE 6 (RDR6) (*light purple*), DICER-LIKE 2 (DCL2) (*light orange*), DICER-LIKE 4 (DCL4) (*pink*), and ssRNAs. RDR6 converts ssRNA into dsRNA, which is then processed by DCL2 and DCL4 into the final easiRNA.

miRNAs are small, noncoding RNAs that negatively regulate gene expression at the post-transcriptional level (72, 73). Their biogenesis involves a two-step cleavage process: first, the primary transcripts (pri-miRNAs) are processed to generate stem loop–structured precursor miRNAs (pre-miRNAs), which are then further cleaved to produce mature miRNA/∗ duplexes (72, 73). In plants, this entire processing occurs in the nucleus and requires the RNase III enzyme DICER-LIKE 1 (DCL1), which operates in conjunction with the double-stranded RNA-binding protein HYPONASTIC LEAVES 1 (HYL1) and the zinc-finger protein SERRATE (SE). These proteins assemble into nuclear granules known as Dicing bodies (D-bodies), which specialize in miRNA processing (Fig. 3*A*) (72, 73). One strand of the duplex, termed the guide strand, is loaded into the miRNA-induced silencing complex, where it directs sequence-specific silencing by binding to complementary target mRNAs, resulting in mRNA cleavage or translational repression (72, 73).

A recent study indicated that DCL1, HYL1, and SE assemble into nuclear D-bodies through LLPS (74). Among these proteins, SE is the primary phase-separating component. SE condensates recruit DCL1, HYL1, and pre-miRNAs, enabling their conversion into mature miRNA/∗ duplexes (Fig. 3*A*) (74). Deletion of SE IDR1 impairs its phase-separation ability, compromising miRNA processing *in vitro* and *in vivo* (74). This highlights SE-driven LLPS as a fundamental mechanism underlying D-body assembly, supporting efficient miRNA biogenesis (Fig. 3*A*).

The function of SERRATE (SE) is tightly regulated by interactions with various binding partners, including a subset of RNA helicases (RH6, RH8, and RH12) (75), the peptidyl-prolyl isomerase Cyclophilin 71 (CYP71) (76), and the IDPs SAID1 and SAID2 (77). The RNA helicases and CYP71 enhance SE phase separation, promoting D-body formation and pre-miRNA processing (75, 76). For CYP71, this regulatory effect relies on its peptidyl-prolyl isomerase activity, as enzymatically inactive CYP71 mutants exhibit a reduced capacity to induce D-body formation and miRNA processing (76). In contrast, SAID1 and SAID2 act as repressors of miRNA biogenesis. They increase SE phosphorylation *via* pre-mRNA processing 4 kinase A (PRPK4A), targeting SE for proteasomal degradation (77). In addition, SAID1/2 bind pri-miRNAs with high affinity, sequestering them from SE and preventing their further processing (77). Notably, RNA helicases, CYP71, and SAID1/2 undergo SE-dependent LLPS and co-assemble into D-bodies, albeit with distinct functional outcomes. While RNA helicases and CYP71 stimulate miRNA processing—the latter by promoting the peptidyl–prolyl isomerization of SE in the condensed phase (75, 76)—SAID1/2 suppress miRNA processing, enabling rapid transcriptome reprogramming (77).

Phase separation also integrates miRNA biogenesis with m^6^A RNA methylation through SE interaction with the methyltransferase complex, specifically MTB and FIP37 (78, 79). MTB, an IDP prone to aggregation, is stabilized in SE condensates, maintaining its solubility and enhancing its methyltransferase activity (78, 79). This co-condensation mechanism protects both SE and MTB from proteasomal degradation, demonstrating how phase separation regulates protein stability (78). Concurrently, SE-MTB condensates recruit the Dicing complex to chromatin at *MIRNA* loci, promoting the cotranscriptional processing of pri-miRNAs (78, 79). SE enhances the methyltransferase complex–mediated deposition of m^6^A marks on single-stranded basal regions of pri-miRNAs (78, 79). Subsequently, m^6^A readers, such as ECT2, recognize m^6^A-modified pri-miRNAs, delivering them to the tethered Dicing complex for efficient processing (78). Thus, LLPS establishes a mechanistic connection between m^6^A RNA methylation and miRNA biogenesis, enabling precise control over miRNA maturation in plants.

### siRNA processing

siRNAs initiate and maintain the genetic silencing of TEs. Specifically, 24-nucleotide siRNAs mediate RdDM to reinforce TE silencing (80). RNA polymerase IV transcribes ssRNAs from TEs or repetitive sequences, which are converted into dsRNAs by RNA-dependent RNA polymerase 2 (RDR2). These dsRNAs are processed into 24-nt siRNAs by DICER-LIKE 3 (DCL3), and the resulting siRNAs are loaded onto Argonaute 4 (AGO4) to form the RNA-induced silencing complex (RISC). The RISC directs siRNAs to their target loci, recruiting factors such as RNA-directed DNA methylation 1 (RDM1) and domains rearranged methyltransferase 2 (DRM2), leading to DNA methylation and TE silencing (80). The biogenesis of siRNAs takes place in nuclear Cajal bodies, which are specialized biomolecular condensates that optimize RdDM efficiency (80).

Upon transcriptional activation, TEs give rise to 21-nt or 22-nt siRNAs, known as epigenetically activated small interfering RNAs (easiRNAs), through a distinct biogenesis pathway (80). Suppressor of gene silencing 3 (SGS3) and RNA-dependent RNA polymerase 6 (RDR6) cooperate to convert TE-derived ssRNAs into dsRNAs, which are subsequently processed by DCL2 and DCL4 into easiRNAs (80). The production of easiRNAs represents a feedback inhibition mechanism, as they mediate the post-transcriptional silencing of active TEs and reinforce their epigenetic repression through the RdDM pathway. This processing occurs within specialized cytoplasmic foci called siRNA bodies (80), which are formed through LLPS (Fig. 3*B*) (81, 82).

The assembly of siRNA bodies is driven by the phase separation of SGS3 through its N-terminal PrLD (81, 82). SGS3 condensates recruit key components of the siRNA processing machinery, including RDR6, silencing defective 5 (SDE5), poly(A)-binding protein 4 (PAB4), DICER-LIKE ribonucleases (DCL2 and DCL4), and Argonaute proteins (AGO1 and AGO7) (Fig. 3*B*) (81, 82). In addition, SGS3-targeted RNAs, particularly ssRNAs, facilitate the incorporation of RDR6 into SGS3 condensates, supporting the hypothesis that SGS3 phase separation underlies the formation of siRNA bodies (82). Plant TEs frequently exhibit low translational efficiency, producing truncated mRNAs that are selectively directed to siRNA bodies (81). Moreover, global defects in translation or mRNA decay pathways trigger the assembly of SGS3 condensates, further emphasizing their role as hubs for efficient siRNA biogenesis (82). In these condensates, target ssRNAs, such as truncated or stalled mRNAs, are converted into dsRNAs by RDR6 and subsequently cleaved into siRNAs by DCL2 and DCL4 (Fig. 3*B*), silencing TEs and other repetitive elements to preserve genome stability (81, 82).

## Phase separation in developmental processes

### Flowering

Flowering time control is essential for plant fitness, as plants must integrate environmental signals to flower at the optimal time, ensuring a successful reproductive strategy. This intricate process involves multiple genetic pathways, among which the autonomous, vernalization, and photoperiod pathways play prominent roles (83). Emerging evidence highlights the importance of phase separation in modulating these regulatory pathways, providing new insights into how plants control flowering at the molecular level (84, 85, 86, 87, 88, 89).

The autonomous pathway promotes early flowering by downregulating the expression of *FLOWERING LOCUS C* (*FLC*) independently of light or temperature signals (83, 90). *FLC* encodes a MADS-box transcription factor that inhibits flowering by blocking the expression of flowering-promoting genes, such as *FLOWERING LOCUS T (FT)* and *SUPPRESSOR OF OVEREXPRESSION OF CONSTANS 1* (*SOC1*) (83, 90). Key regulators in the autonomous pathway include FCA, FLD, FLK, FPA, FVE, and FY, which repress *FLC* through various mechanisms, including epigenetic modifications, RNA polymerase II pausing, alternative polyadenylation, and 3′-end processing of the *FLC* antisense transcript *COOLAIR* (83, 90).

**Figure 4:**
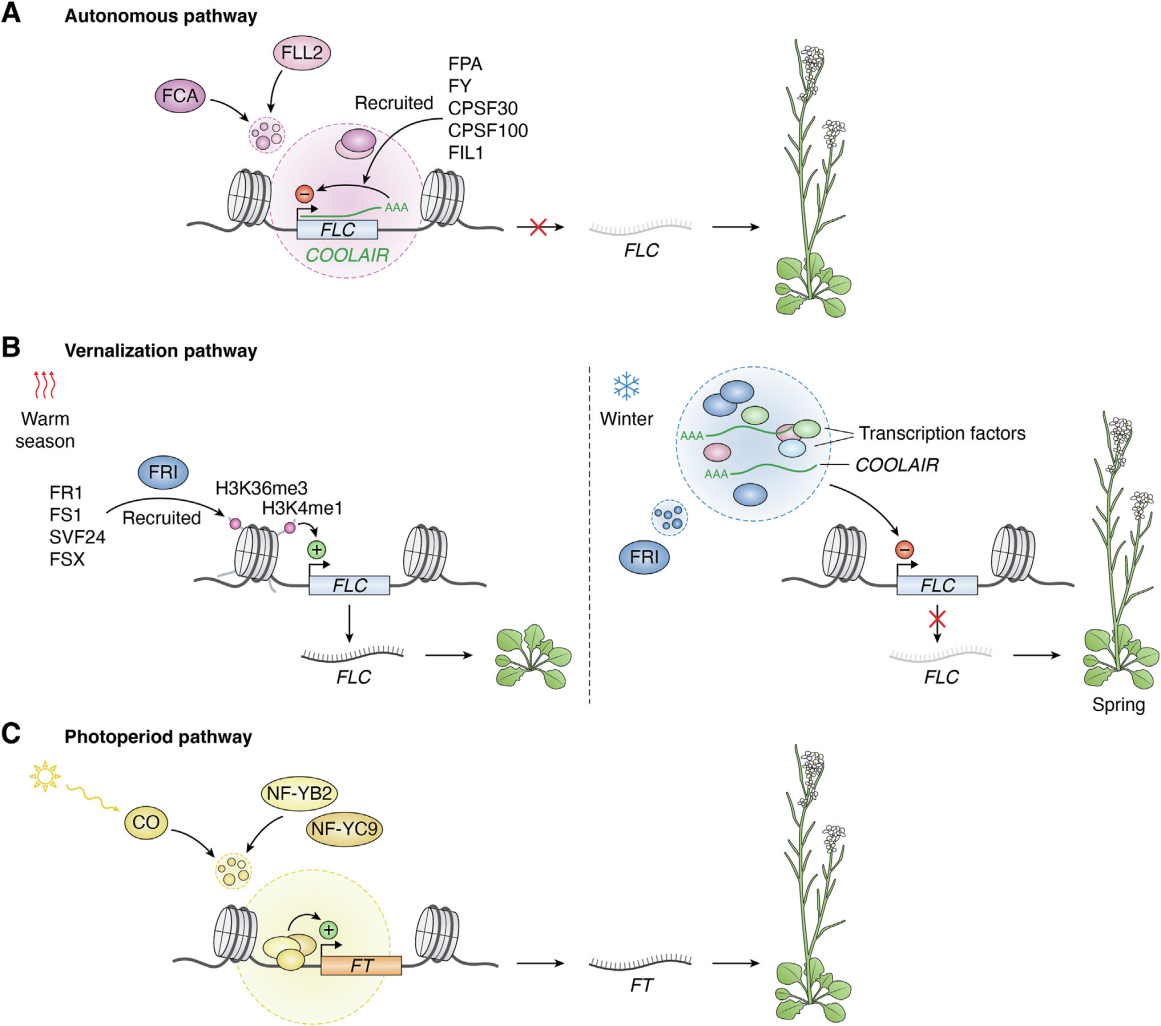
**Phase separation regulation of flowering time in the autonomous, vernalization, and photoperiod pathways.** Schematic representation illustrating the role of phase separation in the regulation of *FLC* and *FT* transcription across different flowering pathways in plants. *A*, *autonomous pathway*. FCA (*dark violet*) and FLL2 (*light violet*) co-condense in the nucleus, forming a molecular scaffold that recruits additional factors, including FPA, FY, CPSF30, CPSF100, and FIL1. This condensate promotes the proximal polyadenylation of the *FLC* antisense transcript *COOLAIR*. The 3′-proximally polyadenylated *COOLAIR* associates with the *FLC* locus, leading to its transcriptional repression. The downregulation of *FLC* facilitates the transition to flowering in spring. *B*, *vernalization pathway*. *Left panel:* During warm seasons, FRIGIDA (FRI) (*blue*) is diffusely distributed in the nucleus, where it recruits proteins such as FR1, FS1, SVF24, and FSX, which facilitate the deposition of transcriptionally active epigenetic marks, including H3K36me3 and H3K4me1 (*pink spheres*), at the *FLC* locus. These marks activate *FLC* transcription, inhibiting flowering. *Right panel:* During winter, FRI (*blue*) forms condensates that sequester transcription factors (*light red* and *green*) together with a distally polyadenylated form of *COOLAIR*, blocking RNA polymerase activity at the *FLC* locus. This repression of *FLC* transcription enables flowering in spring. *C*, *photoperiod pathway*. Upon light stimulation, CONSTANS (CO) (*light orange*) and the nuclear factors NF-YB2 (*yellow*) and NF-YC9 (*dark orange*) form condensates that promote CO binding to the *FT* promoter region. This interaction enhances *FT* transcription, driving flowering.

FCA, a key RNA-binding protein, plays a pivotal role in *COOLAIR* processing by interacting with the 3′-end processing machinery (91, 92). FCA forms nuclear liquid-like granules through LLPS, driven by its N-terminal PrLD (Fig. 4*A*) (84). The coiled-coil protein FLX-LIKE 2 (FLL2) co-condenses with FCA, assembling processing factors, such as FPA, FY, CPSF30, CPSF100, and FIL1, into functional hubs that enhance 3′-end polyadenylation of *COOLAIR* at specific proximal sites (Fig. 4*A*) (84). This proximal polyadenylation resolves an R-loop structure—a three-stranded RNA‒DNA hybrid—formed by *COOLAIR* at the *FLC* locus, triggering chromatin silencing and repressing *FLC* expression, thereby promoting flowering (93).

The vernalization pathway requires prolonged cold exposure to trigger the vegetative-to-reproductive transition. This mechanism ensures that flowering occurs in spring rather than in winter, optimizing conditions for reproductive success (Fig. 4*B*) (83). Under warm temperatures, FRIGIDA (FRI) activates *FLC* expression by recruiting chromatin-modifying complexes that deposit transcriptionally active histone marks, such as H3K36me3 and H3K4me1 (Fig. 4*B*) (94, 95). During prolonged cold exposure, *FLC* expression is repressed through a cotranscriptional mechanism involving *COOLAIR* and epigenetic modifications mediated by the polycomb repressive complex 2 (PRC2) in association with VERNALIZATION INSENSITIVE (VIN) factors (83, 96). *COOLAIR* physically interacts with *FLC* chromatin, facilitating the removal of H3K36me3 and H3K4me1 that were previously deposited under warm conditions (83, 96, 97). Simultaneously, PRC2 catalyzes the deposition of the repressive H3K27me3 mark, which spreads over the *FLC* locus when the temperature increases (83, 96, 98). This vernalization-induced repression of *FLC* is stably maintained through cell division, providing a molecular memory of winter and allowing plants to time flowering to favorable spring conditions (83, 99, 100).

Upon cold exposure, FRI forms nuclear condensates that sequester both FRI and transcriptional activators away from the active *FLC* locus, facilitating *FLC* repression (Fig. 4*B*) (85). FRI condensates depend on *COOLAIR* expression and accumulate a distally polyadenylated isoform of *COOLAIR* (class II) (Fig. 4*B*), potentially linking phase separation to the *COOLAIR*-induced reduction in active histone marks at the *FLC* locus during vernalization (85). In addition to FRI, VRN1, a protein essential for stable *FLC* silencing, undergoes DNA-induced phase separation *in vitro*, driven by charge segregation within its IDRs (101, 102, 103). However, the exact role of VRN1 condensates in *FLC* repression remains unclear.

In mammals, the polycomb-like protein PHF1 forms phase-separated condensates at H3K27me3-marked loci, recruiting PRC2 (104). Furthermore, PRC1, which recognizes H3K27me3 through its CBX subunit, relies on CBX2-mediated phase separation to form PRC1 condensates, promoting chromatin compaction (105, 106). These findings suggest that PRC2-mediated chromatin silencing at the *FLC* locus in plants may similarly involve phase separation mechanisms. Notably, EMBRYO DEFECTIVE 1579 (EMB1579) interacts with MULTIPLE SUPPRESSOR OF IRA4 (MSI4), a homolog of the *Drosophila* nucleosome remodeling factor 55 (p55), as well as DNA damage binding protein 1 (DDB1) (107). Both MSI4 and DDB1 form a complex with CULLIN 4 (CUL4), which, in turn, associates with the CURLY LEAF (CLF)-PRC2 complex to regulate *FLC* expression (107). EMB1579 undergoes LLPS, forming nuclear granules that recruit the MSI4-DDB1-CUL4 complex, which interacts with CLF-PRC2 to regulate H3K27me3 levels and *FLC* repression, ensuring timely flowering (86).

In addition to 3′-end processing and epigenetic events, phase separation influences other transcriptional processes that fine-tune floral transition. The plant hnRNPR-like protein HRLP promotes flowering by inhibiting both splicing and transcription of the *FLC* sense transcript (87). HRLP localizes to nuclear granules, forming LLPS-driven condensates enriched in splicing factors, such as ARGININE/SERINE-RICH 45, regulating *FLC* splicing (87). In addition, HRLP condensates promote R-loop formation, reducing RNA polymerase II enrichment and repressing *FLC* transcription (87). Furthermore, DECAPPING5 (DCP5), a component of PBs, forms LLPS-driven nuclear condensates in partnership with SISTER OF FCA (SSF), binding *FLC* chromatin and reducing RNA polymerase II occupancy, further contributing to *FLC* silencing (88).

In addition to the autonomous and vernalization pathways, phase separation plays a pivotal role in regulating the photoperiod pathway, which integrates light signals with circadian clock-controlled processes to modulate flowering time (108). CONSTANS (CO), a central regulatory hub, activates *FT* under long-day conditions, thereby promoting floral development (108). Recent findings suggest that CO’s transcriptional regulation involves phase separation (Fig. 4*C*) (89). Following extended daylight exposure, CO accumulates in the nucleus, where it assembles into clusters (89). NUCLEAR FACTOR Y SUBUNIT B2 (NF-YB2) and C9 (NF-YC9) shift CO assemblies from solid aggregates to spherical condensates, highlighting their essential role in maintaining the liquid-like properties of CO condensates (89). Importantly, liquid-like CO/NF-YB2/NF-YC9 condensates are highly functional, significantly enhancing *FT* transcription (Fig. 4*C*) (89). The ability of CO to form functional co-condensates with NF-YB2 and NF-YC9 demonstrates a phase separation mechanism that directly links the biophysical properties of condensates to transcriptional activation, enabling precise control of flowering time.

Phase separation also influences flowering time independently of the FT protein and its homologs, known as florigens. In tomatoes, the transcription factor TERMINATING FLOWER (TMF) prevents premature shoot apical meristem (SAM) maturation by repressing the F-box gene *ANANTHA* (*AN*), which drives floral transition (109). TMF forms liquid-like condensates *via* its IDRs and is triggered by cysteine oxidation and DNA binding (110). Elevated hydrogen peroxide (H_2_O_2_) levels accumulate during the vegetative state and oxidize TMF cysteine residues, increasing LLPS and repressing *AN* expression (110). Mutations disrupting TMF phase separation increase *AN* expression and induce early flowering, highlighting its role as a reactive oxygen species (ROS) sensor (110). In addition to TMF, the ALOG family includes other proteins known as TMF family members (TFAMs), which synergize with TMF to regulate floral transition (111). Except for TFAM1 and TFAM2, all TFAMs form heterotypic condensates with TMF, further regulating *AN* expression and floral timing (111). This phase separation mechanism, involving multiple paralogs, regulates meristem maturation to tightly control flowering time in tomatoes.

### Carbon fixation

In all photosynthetic organisms, the fixation of CO_2_ is catalyzed by RuBisCO, an enzyme known for its slow kinetics and susceptibility to errors (112). To mitigate these limitations, eukaryotic microalgae saturate RuBisCO with CO_2_ by sequestering the enzyme into a membraneless granule in the chloroplast stroma, known as the pyrenoid (113). The pyrenoid of the model alga *Chlamydomonas reinhardtii* exhibits characteristics consistent with liquid-like condensates formed through LLPS, including a spherical morphology, fusion and fission events, and rapid fluorescence recovery in fluorescence recovery after photobleaching experiments (114). A key component of the algal pyrenoid is essential pyrenoid component 1 (EPYC1), an intrinsically disordered, repeat-containing protein (115). Mutants lacking EPYC1 exhibit reduced pyrenoid formation and require elevated CO_2_ levels for photoautotrophic growth, indicating that EPYC1 is critical for efficient CO_2_ fixation (115). Reconstitution of the pyrenoid *in vitro* from its isolated minimal components, RuBisCO and EPYC1, has enabled detailed biochemical characterization. Mixing EPYC1 with RuBisCO results in the formation of liquid-like condensates (116). Notably, EPYC1 does not phase-separate independently; instead, it requires RuBisCO to establish multivalent interactions, supporting a codependent network model of LLPS (116). Phase-separated RuBisCO retains enzymatic activity comparable to that of its dispersed form, suggesting that the primary function of the pyrenoid is to enhance CO_2_ availability rather than to improve RuBisCO’s catalytic efficiency (116). The liquid nature of the pyrenoid facilitates the dynamic organization of RuBisCO, enabling efficient CO_2_ fixation under fluctuating environmental conditions.

In C3 plants, which utilize the C3 photosynthetic pathway, RuBisCO catalyzes the fixation of CO_2_ into the three-carbon compound 3-phosphoglycerate during the Calvin cycle (117). However, RuBisCO functions under suboptimal CO_2_ concentrations, reaching only half-saturation with its substrate, which reduces carbon fixation efficiency and increases susceptibility to photorespiration (112). Engineering the pyrenoid into C3 crops could significantly increase CO_2_ fixation and biomass yield (118). EPYC1 directly interacts with the small subunit of RuBisCO from *C. reinhardtii* (119, 120). Initial attempts to express EPYC1 in *Arabidopsis thaliana* failed to induce pyrenoid formation and resulted in EPYC1 degradation (119). Successful pyrenoid formation was achieved by expressing a mature form of EPYC1 together with the *C. reinhardtii* RuBisCO small subunit in the *A. thaliana 1a3b* mutant line, which lacks the endogenous small subunit (121). This led to the formation of a large liquid-like condensate in the chloroplast, which is consistent with pyrenoid assembly (121). Despite this breakthrough, the engineered plants displayed photoautotrophic growth comparable to that of WT plants, indicating that pyrenoid formation alone did not increase photosynthetic efficiency (121). This pioneering work marks a critical step in engineering phase-separated MLOs in plants to improve desirable traits, such as CO_2_ fixation and growth. Future efforts will focus on anchoring the pyrenoid condensate to the thylakoid membrane to facilitate efficient delivery of bicarbonate, a key factor for optimizing pyrenoid function in C3 photosynthetic systems (118, 121). Such advancements could pave the way for significant improvements in plant productivity and growth.

### Light signaling

Light is a critical environmental signal that synchronizes the internal circadian rhythms of plants with external day‒night cycles, optimizing essential physiological processes such as photosynthesis and growth. Photoreceptors, including cryptochromes (CRYs) and phytochromes, sense light and regulate the expression and activity of core components of the molecular oscillator, thereby aligning internal rhythms with light conditions (122, 123).

CRYs are blue-light photoreceptors structurally related to photolyases that play critical roles in plant responses to light (124). In darkness, CRYs exist as inactive monomers, but upon blue light absorption, they undergo conformational changes, promoting oligomerization and interactions with various binding partners (124). Through these complexes, CRYs modulate the expression and stability of light-responsive proteins, regulating plant growth and development (124).

**Figure 5:**
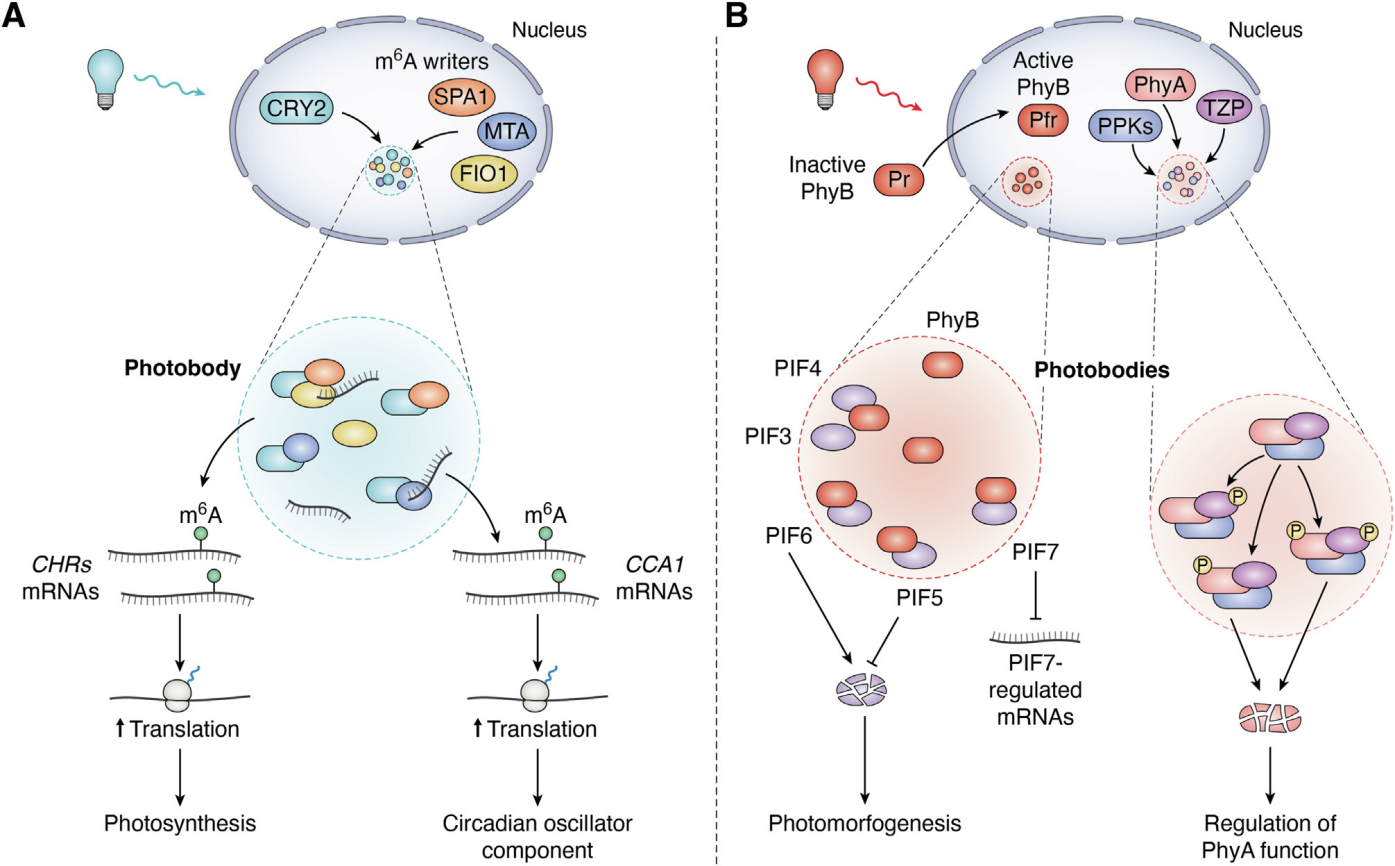
**Light-induced phase separation of cryptochromes and phytochromes into photobodies.** Schematic representation of the mechanism by which light stimuli trigger the phase transition of cryptochrome 2 (CRY2) and phytochromes A and B (PhyA and PhyB) into photobodies. *A*, *CRY2-mediated photobody formation*. Upon blue light absorption, CRY2 (*dark cyan*) interacts with m^6^A writers, including MTA (*blue*) and FIO1 (*yellow*), undergoing phase separation to form photobodies that sequester mRNAs. CRY2 (*dark cyan*)-MTA (*blue*) condensates deposit m^6^A methylation onto *CCA1* mRNA, increasing its translation and regulating the circadian rhythm. Similarly, CRY2 (*dark cyan*)-FIO1 (*yellow*)-SPA1 (*orange*) condensates deposit m^6^A methylation onto *CHR* mRNAs, facilitating their translation and contributing to photosynthesis. *B*, *phytochrome-mediated photobody formation*. In its inactive Pr state, PhyB (*dark red*) resides in the cytoplasm. Absorption of *red* light converts PhyB (*dark red*) to its active Pfr state, triggering its relocation to the nucleus, where it undergoes phase separation to form photobodies. PhyB condensates recruit PIF transcription factors (*light violet*), which promote skotomorphogenesis, although with distinct effects. The interaction of photoactivated PhyB with PIF6 leads to PIF6 degradation, thereby promoting photomorphogenesis. Conversely, sequestration of PIF5 by PhyB photobodies protects it from degradation, whereas sequestration of PIF7 inhibits its chromatin binding and the transcription of PIF7 target genes. In addition, upon *far*-*red* light absorption, PhyA (*light red*) interacts with TZP (*purple*) to form condensates that recruit PPKs (*blue*). These kinases phosphorylate both PhyA and TZP. PhyA phosphorylation targets it for degradation, fine-tuning its regulatory functions.

CRYs mediate blue-light regulation through m^6^A RNA methylation (63, 124). CRY2 interacts directly with all METLL3/14-type m^6^A writers—MTA, MTB, and FIP37, forming nuclear granules that colocalize with CRY2 photobodies (63). These granules exhibit properties of phase-separated condensates, suggesting their formation through LLPS (Fig. 5*A*) (63). The *cry1cry2* mutant shows reduced m^6^A methylation, notably on CCA1 mRNA, encoding a core component of the circadian oscillator (63). This reduction increases CCA1 mRNA degradation, lengthening the circadian period (63). Co-condensation of the CRY2–MTA complex in photobodies promotes m^6^A RNA methylation, stabilizing CCA1 mRNA and maintaining the circadian rhythm under blue light (Fig. 5*A*) (63).

Beyond circadian regulation, CRY2 also partners with FIONA1 (FIO1), a METTL16-type m^6^A writer, to regulate chlorophyll homeostasis (64). In response to blue light, the CRY2–FIO1 complex undergoes phase separation, enhancing m^6^A methylation and translation of mRNAs encoding chlorophyll homeostasis regulators (Fig. 5*A*) (64). This process requires the chaperone protein SUPPRESSOR OF PHYTOCHROME A (SPA1), which recruits FIO1 to CRY2 photobodies, facilitating efficient photosynthesis (Fig. 5*A*) (64).

In addition, CRY condensates concentrate the MOS4-associated complex subunits 3A and 3B (MAC3A/3B), which antagonize ELONGATED HYPOCOTYL 5 (HY5), a key photomorphogenesis regulator (125). Blue light triggers CRY-MAC3A co-condensation, increasing MAC3A chromatin binding, counteracting HY5 activity, and inducing hypocotyl elongation (125). These findings highlight the role of photoinduced phase separation of CRYs in integrating light signals into plant developmental processes.

Phytochromes are photoreceptors that regulate plant growth and development in response to light signals (126). Phytochrome B (PhyB), a primary red/far-red light photoreceptor, undergoes a light-induced conformational switch from its inactive (Pr) form to its active (Pfr) form upon red light absorption (126, 127). In its active state, PhyB interacts with phytochrome-interacting factors (PIFs), a group of basic helix-loop-helix transcription factors that promote skotomorphogenesis, the developmental program enabling plant growth in darkness (126). Red light exposure induces PhyB binding to PIFs, triggering their phosphorylation and subsequent degradation, thereby promoting photomorphogenesis (126). In darkness, PhyB reverts to its inactive Pr state, a process known as dark reversion (126).

In the absence of light, PhyB is diffusely localized in the cytoplasm (126, 128). However, upon red light exposure, it translocates to the nucleus and forms dynamic subnuclear granules known as photobodies (Fig. 5*B*) (126, 128). These photobodies exhibit characteristics of biomolecular condensates, suggesting their formation *via* LLPS (129, 130). PhyB photobodies sequester various PIFs, including PIF3 (129), PIF4 (129), PIF5 (131), PIF6 (130), and PIF7 (132), compartmentalizing PIFs to modulate PhyB-mediated signaling (Fig. 5*B*). Specifically, PhyB photobody formation regulates PIF7 DNA-binding activity, repressing PIF7-target genes in the presence of light and allowing their expression in darkness (Fig. 5*B*) (132). In addition, PhyB photobodies stabilize PIF5 by preventing its degradation in the nucleoplasm, likely enabling plants to fine-tune PIF5 levels in response to environmental light fluctuations, supporting physiological adaptation (Fig. 5*B*) (131).

Phase separation also plays a role in the regulation of phytochrome phosphorylation. Unlike PhyB, PhyA is specifically responsive to far-red light (126, 133). Photoregulatory protein kinases (PPKs) directly phosphorylate PhyA, a process facilitated by the phase-separating protein TANDEM ZINC-FINGER/PLUS3 (TZP) (134). Upon far-red light exposure, TZP and PhyA co-condense into photobodies that recruit PPKs, enhancing PhyA phosphorylation (Fig. 5*B*) (134). Phosphorylated PhyA is subsequently targeted by the COP1/SPA E3 ubiquitin ligase complex for degradation (Fig. 5*B*) (135). By sequestering PPKs, TZP-PhyA photobodies modulate kinase activity, allowing precise regulation of PhyA signaling in response to far-red light (Fig. 5*B*).

## Phase separation in stress response

### Heat

High temperature is a major environmental stress that adversely affects plant growth and development, resulting in significant losses in crop yield and quality (50). The increasing frequency of heat waves due to global warming poses a serious threat to agriculture, jeopardizing global food production (50). To mitigate heat stress, plants have evolved a complex and highly regulated gene network that enables rapid responses to elevated temperatures (136). A deeper understanding of the molecular mechanisms underlying heat stress responses is crucial for the development of heat-resistant crops, ensuring food security in the face of climate change.

Plants exhibit distinct responses to different temperature ranges. Mild, warm temperatures activate a developmental process known as thermomorphogenesis, which includes hypocotyl elongation and flowering alteration (136, 137). In contrast, elevated temperatures induce heat stress, leading to growth and developmental defects that may culminate in cellular damage or death (136). To cope with heat stress, plants have developed sophisticated mechanisms, among which thermosensing plays a key role. Thermosensing enables plants to detect ambient temperature and convert it into intracellular signals, driving physiological adaptation (136, 138).

The evening complex (EC), a central component of the plant circadian clock, functions as a transcriptional repressor that modulates growth in response to temperature fluctuations (139, 140). The EC is composed of three proteins: EARLY FLOWERING 3 (ELF3), which contains a PrLD; the small helical protein EARLY FLOWERING 4 (ELF4); and the DNA-binding protein LUX ARRHYTHMO (LUX) (139). ELF3 regulates plant growth and flowering time in response to elevated temperatures (141). Its PrLD is essential for this function, as replacing it with homologous domains from temperature-adapted plants or truncating its polyglutamine region abolishes temperature responsiveness (142). Under elevated temperatures, ELF3 undergoes PrLD-driven phase separation, forming nuclear granules that sequester the EC and prevent its interaction with chromatin (142). This sequestration influences downstream transcriptional processes, leading to phenotypic outcomes such as hypocotyl elongation and early flowering (142). Consequently, ELF3 acts as a thermosensor in *A. thaliana.* Through ELF3 LLPS, plants can effectively modulate growth and developmental processes in response to temperature changes (142).

In addition to ELF3, the phase behavior of the red/far-red light photoreceptor PhyB is also temperature-dependent (129). Unlike ELF3, PhyB forms phase-separated photobodies in the nucleus at low temperatures, which disassemble at higher temperatures (129). In WT plants, hypocotyl elongation is suppressed at low temperatures and induced at high temperatures, whereas *phyB* mutants exhibit elongation even at low temperatures (129). The expression of PhyB restores the WT phenotype, indicating that PhyB phase separation at low temperatures is essential for thermomorphogenesis (129). Thus, PhyB functions as both a light and a temperature sensor in *A. thaliana*. Similar to the effect of darkness, heat dissolves PhyB condensates, which are crucial for regulating plant growth (126, 129). By compartmentalizing the transcriptional machinery, phase-separated PhyB photobodies coordinate transcriptional and physiological responses to environmental cues, such as light and temperature (129).

A fundamental aspect of the heat stress response is the induction of heat shock proteins (HSPs), a group of molecular chaperones that prevent protein denaturation and aggregation under elevated temperatures (143). This process is driven by heat shock transcription factors (HSFs), which orchestrate genome-wide transcriptional reprogramming to activate diverse protective mechanisms (144). In mammals, HSF1 forms nuclear condensates *via* LLPS, facilitating the transcriptional activation of HSP target genes (145). Heat stress also induces global changes in translational efficiency, primarily through the formation of SGs, which are MLOs comprising nontranslating mRNAs, translation initiation factors, and RNA-binding proteins (5, 146). Although SG formation is a conserved eukaryotic response to various stress stimuli, their structure and function in plants remain poorly understood. Several RNA-binding proteins, such as PABs and RNA-binding protein 47 (Rbp47), serve as markers for SGs in plants (146). Notably, tudor staphylococcal nuclease 1 (TSN1) and TSN2 contribute to thermotolerance by acting as scaffolds for SG assembly (146, 147). SGs are known to form through LLPS (5), underscoring the importance of phase separation as a core mechanism by which proteins and their RNA complexes modulate plant stress responses.

**Figure 6:**
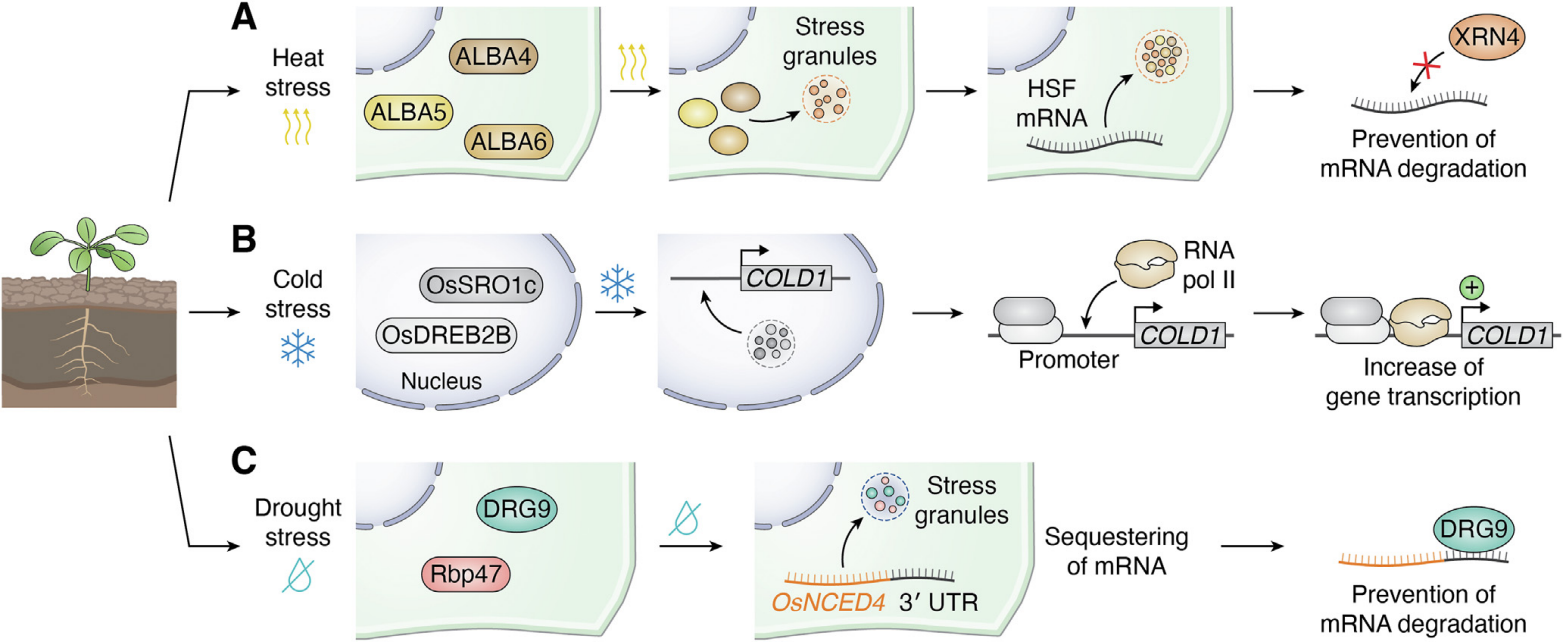
**Phase separation mechanisms involved in the abiotic stress response in plants.** Schematic representation illustrating examples of the molecular mechanisms that utilize LLPS to mediate environmental stress responses in plants. *A*, *heat stress (top row)*. Upon exposure to heat stress (curved arrows icon), ALBA proteins—ALBA4 (*brown*), ALBA5 (*yellow*), and ALBA6 (*orange*)—undergo phase separation, relocating to stress granules (SGs). This redistribution enables ALBA proteins to sequester heat shock transcription factor (HSF) mRNAs, protecting them from XRN4-mediated degradation and promoting heat tolerance. *B*, *cold stress (middle row)*. During cold stress (snowflake icon), OsSRO1c (*dark gray*) co-condenses with the transcription factor OsDREB2B (*light gray*) in the nucleus. This interaction facilitates the binding of OsDREB2B to the *COLD1* gene promoter, increasing its transcription and contributing to cold tolerance. *C*, *drought stress (bottom row)*. Under drought conditions (crossed drop icon), DRG9 (*dark cyan*) undergoes phase separation and translocates to SGs, where it colocalizes with Rbp47 (*red*). This relocation allows DRG9 to sequester OsNCED4 mRNA within SGs, protecting it from degradation and enhancing drought tolerance.

Heat stress triggers phase separation in several plant proteins, facilitating their translocation into liquid-like cytoplasmic granules, primarily SGs (148, 149, 150, 151). This process plays a pivotal role in stabilizing key mRNAs, particularly those encoding HSFs and HSPs, thereby contributing to thermotolerance (148, 149, 150, 151). Among the proteins that undergo heat stress–induced phase separation, the acetylation lowers binding affinity (ALBA) proteins (Fig. 6) (148), RNA-binding glycine-rich group D (RBGD) 2 and RBGD4 (149), glycine-rich protein 7 (GRP7) (150), and Argonaute 1 (AGO1) (151) are key examples. Each of these proteins contributes uniquely to the plant heat stress response, highlighting the versatility of phase separation as a regulatory mechanism.

ALBA proteins are dimeric DNA/RNA-binding proteins essential for genome organization, stability, and RNA translation (152, 153). ALBA4, ALBA5, and ALBA6 contain a conserved ALBA domain followed by a disordered LCD that mediates phase separation under stress conditions (148). During heat stress, ALBA proteins interact with SG and PB components, such as Decapping 5 (DCP5), poly(A)-binding proteins (PAB2, PAB4, PAB8), and Rbp47 (148). Through these interactions, ALBA proteins facilitate the recruitment of specific mRNAs encoding HSFs into SGs (Fig. 6) (148). This sequestration protects these mRNAs from degradation by the exoribonuclease XRN4, increasing their stabilization and contributing to thermotolerance in plants (Fig. 6) (148).

Similarly, RBGD2 and RBGD4, two RNA-binding proteins upregulated in response to heat stress, play crucial roles in thermotolerance through SGs (149). These proteins feature two N-terminal RNA recognition motifs followed by glycine- and tyrosine-rich LCDs, which drive their phase separation (149). Disruption of this property, such as through tyrosine-to-alanine substitutions in the LCD, eliminates their capacity to confer thermotolerance, indicating the importance of phase separation in their function (149). Like ALBA proteins, RBGD2 and RBGD4 interact with SG components, including PAB2, PAB4, PAB8, TSN1, TSN2, and EIF2 GAMMA, under both normal and heat stress conditions (149). During stress, these proteins sequester mRNAs involved in heat and oxidative stress responses, such as those encoding HSFs, HSPs, copper homeostasis proteins, and glutathione S-transferases, into SGs, preserving their stability (149).

GRP7, another RNA-binding protein, comprises an N-terminal RNA recognition motif and a glycine-rich LCD (154). Although GRP7 is better known for its role in cold stress response (154), recent studies have demonstrated its involvement in heat stress adaptation through LLPS (150). A GRP7 variant lacking the C-terminal LCD fails to restore thermotolerance in *grp7* mutants, suggesting that phase separation is essential for its function (150). Under heat stress, GRP7 recruits mRNAs, the translation initiation factor eIF4, and cold-shock proteins (CSP1 and CSP3) into SGs (150). This sequestration inhibits the translation of heat-responsive mRNAs, including those encoding GRP7 itself and HSFs, facilitating heat stress adaptation (150).

Argonaute1 (AGO1), a core component of the RISC (80), also undergoes phase separation under heat stress, driven by its N-terminal PrLD (151). AGO1 interacts with several SG components, including PABP2, Rbp47, RNA helicases (RH6, RH8, RH11, RH52, RH53), G3BP1, and TSN1/2 (151). In addition, AGO1 associates with PB components and proteins involved in mRNA decay, such as DCP5, VARICOSE, La-related protein 1a (LARP1a), and UPFRAMESHIFT1 (UPF1) (151). Notably, while DCP5 is a PB component under normal conditions (146), it colocalizes with PAB2 in SGs during heat stress (151). These findings suggest that heat stress drives AGO1 accumulation in SG condensates, along with siRNA body components and proteins involved in mRNA turnover (151). Despite its relocalization to SGs, the miRNA repertoire and AGO1-miRNA loading are only modestly affected, indicating that AGO1 retains its silencing function (151).

The ability of the ALBA proteins RBGD2/4, GRP7, and AGO1 to undergo heat-induced phase separation and localize to SGs highlights the central role of LLPS in plant stress responses. By sequestering key mRNAs and proteins, these condensates stabilize critical transcripts, protecting them from degradation during stress (148, 149, 150, 151). This guarantees their availability for translation once normal conditions are restored, thereby increasing thermotolerance. Collectively, these findings highlight the versatility of LLPS-mediated mechanisms in plant biology, demonstrating how rapid compartmentalization allows plants to adapt to extreme environmental challenges, resume growth, and maintain development after stress is alleviated.

### Cold

Heat is not the only environmental challenge for crops; climate change is altering weather patterns and exacerbating temperature extremes, making cold stress an increasingly significant concern (155). Recent studies have revealed that LLPS plays a role in plant responses to cold (156, 157), although research in this area remains limited.

One well-characterized example of cold-induced LLPS is CP29A, a chloroplast-specific RNA-binding protein (156). CP29A condensation depends on its PrLD, as deletion of this domain significantly reduces its propensity for phase separation (156). The functional relevance of this LLPS behavior is evident from studies on *cp29a* null mutants, which present leaf rosette defects, impaired chloroplast RNA splicing, and reduced global translation (156). Importantly, these phenotypes are not rescued by a PrLD-deficient CP29A variant, indicating that CP29A phase separation is essential for its function (156). Interestingly, CP29A condensates associate with chloroplast nucleoids, suggesting involvement in co-transcriptional RNA splicing (156). However, CP29A condensation occurs within the typical seasonal temperature range and is not considered a stress response, implying its role in plant adaptation to regular temperature fluctuations (156).

In contrast, OsSRO1c, a member of the Similar to RCD One protein family, exemplifies cold-induced LLPS as a stress response mechanism in rice (157). OsSRO1c forms liquid-like condensates under cold stress conditions, driven primarily by its IDR (157). OsSRO1c interacts with the transcription factor OsDREB2B, increasing its activity through co-condensation (Fig. 6) (157). Functionally, the OsSRO1c–OsDREB2B complex binds to the CHILLING TOLERANCE DIVERGENCE 1 (COLD1) promoter, significantly enhancing its transcription beyond the capacity of OsDREB2B alone (Fig. 6) (157). This amplification of COLD1 transcription highlights the pivotal role of LLPS in mediating cold tolerance, with the OsSRO1c–OsDREB2B complex acting as a molecular amplifier of cold-responsive gene expression (157).

### Drought

Drought, often accompanied by heat stress, is one of the most significant abiotic factors limiting crop yields (158). The accelerating effects of climate change, including rising temperatures and increasing water scarcity, emphasize the urgent need for high-yield, water-efficient crops (159). Drought stress leads to cellular dehydration, osmotic stress, and ROS accumulation (158, 159). In response, plants have evolved physiological and molecular adaptation strategies, including reducing water loss, producing osmolytes, activating antioxidant defenses, and regulating hormonal and transcriptional pathways (158, 159).

A growing body of evidence suggests that phase separation plays a critical role in modulating these drought-induced molecular responses (160, 161, 162, 163, 164). A key example is the dsRNA-binding protein drought resistance gene 9 (DRG9), which was identified as a positive regulator of the drought stress response in rice (160). Under nonstress conditions, DRG9 is diffusely distributed in the cytoplasm (160). However, upon exposure to drought stress, it undergoes phase separation and relocates to SGs, where it colocalizes with Rbp47 (Fig. 6) (160). This relocalization is functionally significant, as DRG9 binds the OsNCED4 mRNA, protecting it from degradation (Fig. 6) (160). OsNCED4 catalyzes the rate-limiting step in ABA biosynthesis (165), a critical pathway in drought adaptation (158, 159). By stabilizing OsNCED4 mRNA, DRG9 enhances ABA accumulation, promoting drought tolerance (160). Thus, DRG9 is a molecular drought sensor that uses phase separation to stabilize stress-responsive mRNAs and enhance the ABA-mediated drought response (160).

The interplay between m^6^A RNA modification and ABA signaling has also emerged as a key mechanism in the drought stress response. ABA rapidly accumulates during drought, activating specific receptors that trigger downstream signaling pathways critical for stress adaptation (158, 159). Two m^6^A readers, SiYTH1 in foxtail millet (*Setaria italica*) (161) and ECT8 in *A. thaliana* (162), are upregulated under drought stress or ABA signaling. Both proteins undergo phase separation, forming dynamic cytoplasmic granules under stress conditions (161, 162).

SiYTH1, a positive regulator of drought tolerance, directly binds m^6^A-modified transcripts involved in ROS scavenging, stomatal closure, and ABA signaling (161). Through phase separation, SiYTH1 stabilizes these transcripts, reducing their degradation and promoting drought tolerance (161). Conversely, ECT8 negatively regulates ABA signaling. Upon drought or ABA exposure, ECT8 sequesters m^6^A-modified mRNAs, including those encoding the ABA receptor PYL7, into SGs (162). This reduces the cytoplasmic availability of PYL7 mRNA, lowering PYL7 protein levels (162). This sequestration provides a negative feedback mechanism to fine-tune ABA sensitivity, preventing excessive ABA responses and ensuring appropriate physiological adaptation to drought stress (162).

One important consequence of drought is osmotic stress, which leads to water loss, cell shrinkage, and reduced growth (166). Osmotic stress decreases cell volume, causing molecular crowding that acts as a stress signal, which is detected by intracellular proteins *via* LLPS (163, 164). Two major osmotic stress sensors, SEUSS (SEU) (163) and DCP5 (164), respond to molecular crowding by forming liquid-like granules through LLPS. While SEU granules localize to the nucleus (163), DCP5 granules form in the cytoplasm (164). Despite their distinct locations, both rely on IDRs for osmosensing (163, 164). SEU phase separation is driven by the compaction of its N-terminal IDR1, which depends on two conserved dynamic α-helices (163). Similarly, DCP5 phase separation is triggered by the compaction of a specific IDR region termed the intramolecular crowding sensor (ICS) (164). This ICS mediates phase separation through multivalent hydrophobic interactions and is unique to land plants, suggesting evolutionary adaptation for plant-specific stress responses (164).

SEU and DCP5 exemplify distinct mechanisms of plant adaptation to osmotic stress through LLPS. SEU nuclear condensates promote the expression of stress-responsive genes, enhancing osmotic stress tolerance (163). Plants expressing SEU variants lacking IDR1 fail to survive under osmotic stress, highlighting the critical role of SEU-mediated phase separation in stress adaptation (163). In contrast, DCP5 cytoplasmic granules are enriched in SG components, including RNA-binding proteins and translation initiation factors (164). However, DCP5 condensates assemble independently of the SG marker G3BP1, suggesting that they represent a distinct form of plant SGs, named DCP5-enriched osmotic stress granules (DOSG) (164). DOSGs selectively sequester mRNAs, reshaping the transcriptome by downregulating growth-related genes while upregulating stress-responsive genes (164). Remarkably, DCP5 variants lacking ICS only partially rescue the *dcp5* mutant defects, emphasizing the importance of LLPS-driven DOSG formation in the osmotic stress response (164). These findings reveal a novel osmosensing and osmotic stress adaptation mechanism in plants based on phase separation, which ensures survival under hyperosmotic conditions.

In addition to their roles in vegetative tissues, phase separation mechanisms influence seed germination under water-limiting conditions. To endure harsh environments, plant seeds remain dormant in a desiccated state (167). In contrast, seedlings are far more vulnerable to environmental fluctuations, particularly water availability. Thus, seed germination is tightly regulated to occur only under favorable conditions (167).

In *A. thaliana*, FLOE1, a PrLD-containing IDP, plays a critical role in this process. FLOE1 undergoes reversible, hydration-dependent LLPS in embryos: desiccated embryos lack FLOE1 condensates, whereas hydration induces their formation (168). The *floe1* KO mutant displays increased germination under water-deprived conditions, indicating that FLOE1 suppresses germination during water scarcity (168). The amino acid sequence and phase behavior of FLOE1 vary widely across plant species, suggesting evolutionary adaptation of seed germination to local water conditions (168). By modulating germination through dynamic condensation, FLOE1 acts as a water sensor, enhancing seedling survival in fluctuating environments (168).

### Salinity

Salt stress represents a significant challenge to agriculture, reducing crop yield and quality. It currently impacts nearly 40% of irrigated farmland, accelerating soil degradation and decreasing the availability of arable land (169, 170). Salt stress disrupts osmotic and ionic balance, reducing water uptake, causing ion toxicity, and triggering oxidative stress. This negatively affects growth, photosynthesis, and metabolic processes (169, 170). In response, plants activate signaling pathways that regulate ion transport, produce osmoprotectants (*e.g.*, proline), and initiate hormonal responses, particularly through ABA, to modulate gene expression and promote stomatal closure (169, 170). As soil salinization continues to increase due to improper irrigation practices and climate change, global food security is becoming increasingly at risk (169, 170). Consequently, understanding the mechanisms of salt tolerance in plants is crucial for developing salt-tolerant crops and mitigating the adverse effects of salinity on agriculture.

Emerging research highlights phase separation as a critical mechanism in plant responses to salt stress, offering new insights into cellular adaptation strategies (171, 172). The transcription factor SHOOT MERISTEMLESS (STM) plays a crucial role in maintaining the SAM by preventing cellular differentiation (173). STM undergoes LLPS through its PrLD, forming nuclear condensates that enhance its meristem maintenance function (171). STM condensates facilitate its interaction with the Mediator complex subunit MED8, upregulating the expression of STM target genes (171). Notably, salt stress promotes STM condensation, increasing its transcriptional activity and stimulating shoot branching, an adaptive response that provides additional growth axes and enhances plant survival under high salinity (171). Thus, STM phase separation represents a dynamic mechanism by which plants sense and respond to salt stress, adjusting their developmental programs to increase resilience (171).

In addition to intracellular phase separation, extracellular LLPS has emerged as a key component of plant responses to salt stress. FERONIA receptor kinase (FER) forms a signaling complex with its co-receptor LORELEI-LIKE glycosylphosphatidylinositol-anchored protein 1 (LLG1), which is activated by its peptide ligand rapid alkalinization factor (RALF) (174). Pectin, a major cell wall polysaccharide, particularly its de-esterified forms and pectin oligosaccharides, interacts with RALF at the plasma membrane‒cell wall interface, driving phase separation and forming pectin‒RALF condensates (172). These condensates cluster FER-LLG1 and other noncognate receptors, inducing inward membrane bending and enhancing receptor endocytosis (172).

This pectin-RALF-FER-LLG1–driven endocytosis is a critical adaptive response to environmental stress. Under salt and heat stress, elevated levels of RALF1 and de-esterified pectin promote the formation of RALF-pectin condensates and the clustering of FER–LLG1 complexes (172). This process amplifies receptor endocytosis and activates multiple signaling pathways, facilitating rapid stress responses (172). Inhibition of RALF-pectin LLPS–mediated receptor endocytosis significantly reduces seedling growth under salt and heat stress, underscoring its importance in stress tolerance (172). Unlike previously characterized intracellular phase separation mechanisms, this extracellular process involves interactions between cell wall polymers and secreted peptides, functioning as a novel signaling platform for environmental adaptation.

### Nutrient deficiency

Nitrogen deficiency in plants leads to impaired growth, reduced chlorophyll content, and decreased photosynthetic efficiency, resulting in stunted development and lower crop yields (175). As a vital macronutrient, nitrogen is essential for the synthesis of amino acids, nucleotides, and pigments (175). In agricultural systems, nitrogen is often replenished through chemical fertilizers; however, such practices contribute to groundwater contamination and eutrophication (175). Understanding the mechanisms by which plants respond to nitrogen deficiency is crucial for developing sustainable agricultural strategies that enhance nutrient uptake, improve nitrogen use efficiency, and minimize dependence on synthetic fertilizers, thereby reducing environmental impact.

ORESARA1 (ORE1) is a key transcription factor that regulates nitrogen deficiency–induced senescence (176). ORE1 interacts directly with the intrinsically disordered Mediator subunit MED19a, driving the senescence response under nitrogen-limited conditions (177). In response to nitrogen deficiency, MED19a undergoes LLPS *via* its C-terminal charged IDR, forming nuclear condensates that recruit ORE1 (177). These MED19a–ORE1 condensates promote the transcriptional activation of ORE1 target genes, which act as effectors of cellular senescence (177). These findings pinpoint the critical role of phase separation as a dynamic mechanism that enables plants to adapt rapidly to nutrient deprivation.

## Conclusions and future perspectives

The discovery of biomolecular condensates formed *via* LLPS has fundamentally transformed our understanding of cellular organization, highlighting their critical roles in gene expression regulation (47, 48, 49), stress adaptation (22, 40, 41, 42), and disease pathology (17, 21, 22). In animal cells, LLPS governs the formation of SGs (5) and PBs (6), which regulate mRNA metabolism by controlling the balance between mRNA storage, degradation, and translation (5, 6). The nucleolus, the largest and best-characterized phase-separated condensate, is essential for ribosome biogenesis, orchestrating the spatial compartmentalization of RNA and proteins to facilitate ribosomal RNA processing and assembly (8). LLPS also plays a crucial role in chromatin organization, as exemplified by heterochromatin foci, where the heterochromatin protein 1α phase separates to maintain transcriptional repression (37, 38). Similarly, super-enhancer condensates drive transcriptional regulation by clustering Mediator, BRD4, and other transcriptional activators to enhance gene expression (178). LLPS dysregulation has been implicated in various diseases, including neurodegenerative disorders such as amyotrophic lateral sclerosis and frontotemporal dementia, where aberrant phase separation of RNA-binding proteins such as FUS (21) and TDP-43 (179) leads to cytotoxic aggregation. Furthermore, LLPS dysfunction contributes to cancer progression, with proteins such as the tumor suppressor p53 (180) and the transcriptional regulator SPOP (181) undergoing phase separation to modulate tumorigenesis.

While the fundamental molecular principles of LLPS—multivalent interactions, IDRs, and nucleic acid binding—are conserved between animal and plant cells, key differences arise due to the distinct cellular architecture and environmental constraints of plants. Like their animal counterparts, plant cells contain SGs and PBs involved in RNA metabolism (146, 182), as well as heterochromatin condensates that regulate chromatin organization (55). However, differences in the composition of these condensates reflect evolutionary divergence. Mammalian SGs are primarily scaffolded by Ras-GAP SH3 domain-binding protein (G3BP1/2), T-cell intracellular antigen-1, and T-cell intracellular antigen-related protein (5, 182), whereas plant SGs rely on oligouridylate-binding protein 1, Rbp47, and TSN1/2 as structural components (146, 182). Similarly, plant heterochromatin condensates form through the action of Agenet domain-containing protein 1 (ADCP1), a functional analog of mammalian heterochromatin protein 1α with no sequence similarity (55). LLPS also regulates transcription in plants, as observed in the phase separation of TMF transcription factors, which coordinate floral development (110, 111), and the co-condensation of STM with the Mediator subunit MED8, which governs SAM differentiation (171).

In addition to these conserved condensates, plants exhibit unique LLPS-driven MLOs tailored to their specific physiological demands. Dicing bodies (D-bodies), for instance, are condensates involved in microRNA biogenesis, assembled around DCL1 and other RNA-processing factors (74). These structures, which facilitate pri-miRNA processing, are absent in animals (74). Another plant-specific condensate, the photobody, is a nuclear compartment that regulates light signaling by concentrating photoreceptors such as PhyB (129, 130). In algae, LLPS drives the formation of the pyrenoid, an organelle enriched in the enzyme RuBisCO and the scaffold protein EPYC1, which enhances CO_2_ fixation efficiency and optimizes photosynthesis (114, 115, 116). LLPS also mediates key stress responses in plants, as exemplified by RALF-pectin condensates, which assemble extracellularly at the plasma membrane–cell wall interface to integrate mechanical and hormonal signals in response to salt and heat stress (172). These plant-specific condensates underscore the evolutionary divergence of LLPS functions between plants and animals, with plant LLPS predominantly facilitating adaptive responses to environmental challenges and regulating developmental plasticity.

As sessile organisms perpetually subjected to environmental variability, plants employ LLPS as a dynamic and reversible mechanism to rapidly perceive and adapt to stress. Stress conditions can either induce the formation of biomolecular condensates or trigger their disassembly, both of which serve as critical mechanisms for physiological adaptation. In the nucleus, LLPS modulates transcription by either sequestering components of the transcriptional machinery or enhancing their interaction with chromatin. For example, heat stress induces condensation of ELF3, which sequesters the EC away from chromatin, resulting in transcriptional reprogramming that promotes hypocotyl elongation and early flowering (142). Under nitrogen deficiency, ORE1 co-condenses with the Mediator subunit MED19a, facilitating the transcriptional activation of senescence-associated genes (177). Conversely, heat stress disrupts phase-separated PhyB photobodies, releasing PIFs and increasing their transcriptional activity, which in turn promotes hypocotyl elongation (129). In the cytoplasm, LLPS primarily governs mRNA metabolism by sequestering specific transcripts and directing them toward storage or degradation. SGs and PBs are key biomolecular condensates that are central to this regulatory process (5, 6, 146, 182). PBs, which are constitutively present, increase in size and number under stress, promoting mRNA decay (6, 146, 182). In contrast, SGs form transiently during stress, stabilizing specific transcripts for future translation upon stress recovery (5, 146, 182). The composition of these condensates dynamically changes in response to stress, reflecting the cellular need for precise translational control (182). Several LLPS-prone proteins exhibit stress-dependent phase separation, transitioning between soluble and condensed states in response to environmental cues. Under heat stress, RNA-binding proteins such as ALBA (148), RBGD2/RBGD4 (149), GRP7 (150), and AGO1 (151) relocalize to SGs, where they protect stress-responsive mRNAs from degradation and regulate their translation. Notably, ALBA proteins relocate to both SGs and PBs specifically under heat stress (148), illustrating how LLPS responses are tailored to distinct stress conditions.

Beyond the formation of condensates, some proteins shuttle between different phase-separated compartments depending on the cellular conditions. For example, DCP5 resides in PBs under normal conditions (146) but translocates to SGs under heat stress (151) and assembles into DOSGs during osmotic stress (164). Since SGs and PBs can exchange components (146, 182), this relocalization likely facilitates functional crosstalk between mRNA decay and storage pathways, ensuring a rapid and coordinated stress response. Furthermore, LLPS-driven sequestration can modulate the availability of key regulatory proteins, thereby antagonizing specific cellular processes. For example, ELF3 condensation under heat stress sequesters the EC (142), potentially inhibiting its role in circadian clock regulation. This underscores how phase separation acts as a regulatory switch, dynamically balancing stress adaptation with the maintenance of normal cellular functions. By leveraging LLPS, plants achieve rapid, reversible, and context-specific stress responses, promoting survival under challenging conditions.

Studying phase separation in plants presents both unique advantages and challenges. Plants are excellent models for investigating stress-related LLPS, providing valuable insights into how biomolecular condensates contribute to adaptive responses. In addition, plant systems offer powerful genetic tools, such as *A. thaliana* mutants and CRISPR-based genome editing, enabling precise manipulation of condensate-forming proteins and their functional characterization. However, compared with metazoans, plant LLPS research remains relatively underexplored. Experimental limitations, including the presence of a rigid cell wall and high autofluorescence in plant tissues, pose significant challenges to live-cell imaging and the visualization of condensate dynamics. Furthermore, while phase-separated condensates in animals are frequently implicated in diseases such as cancer and neurodegeneration, plant condensates have not been linked to pathological conditions in the same manner, potentially limiting their translational relevance.

Despite significant progress, research on plant LLPS remains predominantly focused on biological phenomena, often lacking detailed biophysical and structural characterization of the condensed phase. This gap reflects the interdisciplinary nature of phase separation studies, which require the integration of plant molecular biology with biochemical and biophysical approaches. Future research should aim to elucidate the structural dynamics of biomolecular condensates through a combination of biochemical reconstitution, advanced imaging techniques, and computational simulations. Such efforts are essential for a comprehensive understanding of how phase-separating proteins function in plants under both normal and stress conditions. This knowledge is vital for translating current insights into practical agricultural applications. Harnessing phase separation mechanisms could enable the development of crops with enhanced resilience to environmental stresses, thereby improving agricultural productivity and sustainability in the face of climate change.

## Conflict of interest

The authors declare that they have no conflicts of interest with the contents of this article.
